# Insights Into Immunothrombosis: The Interplay Among Neutrophil Extracellular Trap, von Willebrand Factor, and ADAMTS13

**DOI:** 10.3389/fimmu.2020.610696

**Published:** 2020-12-02

**Authors:** Junxian Yang, Zhiwei Wu, Quan Long, Jiaqi Huang, Tiantian Hong, Wang Liu, Jiangguo Lin

**Affiliations:** ^1^ Research Department of Medical Sciences, Guangdong Provincial People’s Hospital, Guangdong Academy of Medical Sciences, Guangzhou, China; ^2^ Institute of Biomechanics/School of Bioscience and Bioengineering, South China University of Technology, Guangzhou, China

**Keywords:** neutrophil extracellular traps, von Willebrand factor, ADAMTS13, thrombotic microangiopathy, acute ischemic stroke, COVID-19

## Abstract

Both neutrophil extracellular traps (NETs) and von Willebrand factor (VWF) are essential for thrombosis and inflammation. During these processes, a complex series of events, including endothelial activation, NET formation, VWF secretion, and blood cell adhesion, aggregation and activation, occurs in an ordered manner in the vasculature. The adhesive activity of VWF multimers is regulated by a specific metalloprotease ADAMTS13 (a disintegrin and metalloproteinase with thrombospondin type 1 motifs, member 13). Increasing evidence indicates that the interaction between NETs and VWF contributes to arterial and venous thrombosis as well as inflammation. Furthermore, contents released from activated neutrophils or NETs induce the reduction of ADAMTS13 activity, which may occur in both thrombotic microangiopathies (TMAs) and acute ischemic stroke (AIS). Recently, NET is considered as a driver of endothelial damage and immunothrombosis in COVID-19. In addition, the levels of VWF and ADAMTS13 can predict the mortality of COVID-19. In this review, we summarize the biological characteristics and interactions of NETs, VWF, and ADAMTS13, and discuss their roles in TMAs, AIS, and COVID-19. Targeting the NET-VWF axis may be a novel therapeutic strategy for inflammation-associated TMAs, AIS, and COVID-19.

## Introduction

Neutrophils, the most abundant leukocyte subset in circulation, act as the critical responders during innate immunity and inflammation (1). Neutrophils have been known to kill and clear invading microorganisms through two strategies: phagocytosis and degranulation. Brinkmann et al. firstly reported a novel antimicrobial strategy of neutrophils, by which neutrophil extracellular traps (NETs), the net-like chromatin structure decorated with histones and granular proteins, are released from neutrophils into extracellular space to catch and kill invading bacteria to protect host from infection (2). However, NETs have a dark side. Uncontrolled NET formation or improper clearance of NETs may lead to tissue damage and activate inflammatory cells, contributing to the development of multiple diseases, such as fibrosis (3), sepsis (4), cancer metastasis (5), systemic lupus erythematosus (SLE) (3), thrombosis (6), and atherosclerosis (7).

Plasma glycoprotein von Willebrand Factor (VWF), which captures circulating platelets to the sites of vascular injury and mediates subsequent platelet activation and aggregation, is a critical mediator in hemostasis (8). The activity of VWF depends on its size. Ultra-large VWF (UL-VWF) multimers released from endothelial cells may spontaneously recruit excessive circulating platelets and other blood cells, promoting the development of thrombosis (9). Metalloprotease ADAMTS13 (a disintegrin and metalloproteinase with thrombospondin type 1 motifs, member 13) specifically cleaves the Tyr1605-Met1606 bond within VWF A2 domain to regulate the size and activity of VWF multimers, preventing the formation of thrombus (10).

Increasing studies indicate that NETs, like VWF, play an important role in the formation of thrombus in venous (11), arterial (12), and cancer-associated thrombosis (13). NETs directly interact with VWF *via* electrostatic force (14), and this interaction retains NETs on endothelial surface (15). The colocalization of NETs and VWF has been observed in venous (11) and arterial thrombosis (12). Given that both NETs and VWF have prothrombotic and proinflammatory effects, it therefore is reasonable to speculate that the interactions between NETs and VWF may promote the development of thrombosis and inflammation. Moreover, NET contents indirectly or directly reduce the activity of ADAMTS13, promoting the formation of UL-VWF. NETs, VWF, and ADAMTS13 may form a vicious circle to aggravate the development of thrombosis and inflammation. In this review, we will summarize the biological characteristics of NETs, VWF, and ADAMTS13, and describe the interactions among NETs, VWF, and ADAMTS13. We also discuss the latest findings regarding the role of NET-VWF axis in thrombotic microangiopathies (TMAs), acute ischemic stroke (AIS), and coronavirus disease 2019 (COVID-19), and the therapeutic potential by targeting the NET-VWF axis.

## The Mesh-Like Neutrophil Extracellular Trap

### NET Structure and Components

In 2004, NETs were first described as a form of initial immune defense released from neutrophils (2). The network of NETs is mainly composed of DNA, histones and proteins from both azurophilic and specific granules to virulence factors (2). Neutrophil elastase (NE), myeloperoxidase (MPO), cathepsin G (CG), proteinase 3 (PR3), metalloproteinases 9 (MMP-9), and human neutrophil peptides 1 (HNP1) have been illustrated to present in NETs (5, 16, 17). NET-associated proteins provide a high local concentration of antimicrobial agents that is lethal to bacteria. Urban et al. analyzed 15 NET-associated proteins and demonstrated that 10^12^ neutrophils can release NETs containing, on average, 3.58 ± 0.28 g of protein and 2.24 ± 0.51 g of DNA, indicating a ratio of 1.67 ± 0.26 g of protein per gram of DNA (18). Of note, the proportion of composition, protein types and post-translational modifications of NETs induced by distinct stimuli are various (19). Combining fluorescence and atomic force microscopy, Pires et al. demonstrated that NETs were organized as branched networks. The topological height of NETs was 3 ± 1 nm and the area of the pores was up to 0.03 ± 0.04 µm^2^, so that pathogens whose size is within this range can be captured (20).

### Mechanisms of NET Formation

**Figure 1:**
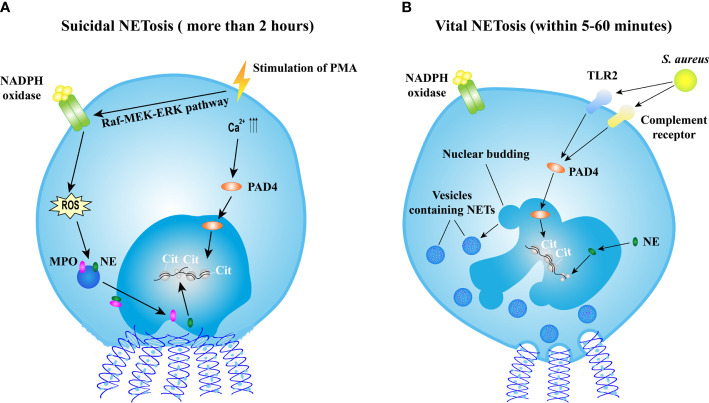
Mechanisms of NET formation. **(A)** The mechanism of suicidal NETosis. Suicidal NETosis induced by PMA takes approximately 2 h. Stimulation of PMA induces the assembly of NADPH oxidase (NOX) *via* the Raf-MEK-ERK pathway, resulting in ROS generation. ROS triggers the activation of MPO and NE, facilitating the translocation of MPO and NE into nucleus. NE promotes the decondensation of chromatin by degrading histones. In addition, the stimulation also increases the levels of intercellular calcium, might result in the activation of PAD4 that subsequently translocates into nucleus and citrullinates histones leading to chromatin decondensation. Thereafter, nucleus rounds up, nucleus envelope ruptures, cell membrane ruptures, and then eventually chromatin decorated with various granular proteins is released into extracellular space and cell is lysed. **(B)** The mechanism of vital NETosis. Vital NETosis is rapid and non-lysis. *S. aureus*-induced vital NET formation requires TLR2 and complement receptor and is independent of NOX-generated ROS. Activated PAD4 and NE induce chromatin decondensation, and then vesicles filled with NETs are generated. These vesicles are expelled from nucleus and released into extracellular space without the disruption of cell membrane. In vital NETosis, cell membrane integrity is maintained and the cell remains functional. NET, neutrophil extracellular trap; NETosis, a process of neutrophil extracellular trap formation; PMA, phorbol myristate acetate; NADPH, nicotinamide adenine dinucleotide phosphate; ROS, reactive oxygen species; MPO, myeloperoxidase; NE, neutrophil elastase; *S. aureus*, *Staphylococcus aureus*; PAD4, protein-arginine deaminase type 4; TLR2, toll-like receptor type 2.

Various stimuli, such as bacteria, fungi, viruses, parasites, activated platelets, and some chemical substances, are able to induce NET formation termed as NETosis (16). Recently, the cellular events of NET formation were demonstrated (21, 22) and extensively reviewed elsewhere (23). Briefly, NET formation is initiated by actin disassembly, followed by plasma membrane microvesicles shedding, vimentin remodeling, microtubule disassembly, endoplasmic reticulum vesiculation, chromatin decondensation, and nuclear rounding, progressively increased plasma membrane and nuclear envelope permeability, nuclear lamin meshwork, and then nuclear envelope rupture to release chromatin into the cytoplasm, and finally plasma membrane rupture and discharge of extracellular chromatin (21). However, the mechanism of NET formation is complicated and still not fully understood. The molecular events leading to NET formation is intensively studied with the stimulation of phorbol 12-myristate 13-acetate (PMA). PMA activates the Raf-MEK-ERK pathway, resulting in the assembly of nicotinamide adenine dinucleotide phosphate (NADPH) oxidase and the generation of reactive oxygen species (ROS). ROS triggers the activation of the azurosome proteases and MPO, allowing these proteases to pass through the intact membrane (24). NE, which functions as an antimicrobial protease, translocates to the nucleus, and drives the degradation of histones inducing chromatin decondensation (25). In addition, the increased levels of intracellular calcium activate peptidylarginine deiminase 4 (PAD4) that converts arginines in histones to citrullines, reducing the positive charge of histones and contributing to chromatin decondensation as well (26). Sequentially, the nucleus loses the characteristic nuclear lobuli and rounds up, nuclear envelope ruptures, cell membrane ruptures, and chromatin is released into extracellular space (22). This process takes more than 2 h, and is termed as suicidal NETosis (**Figure 1A**). Notably, the requirement of NADPH or PAD4 is not universal to all NETotic pathways. For instance, NETosis can be induced in a NADPH oxidase-independent manner (27, 28) or in a PAD4-independent manner (29, 30).

Apart from suicidal NETosis, increasing evidence demonstrates that NETs could be induced in a rapid way. Stimulated by *Staphylococcus aureus* (*S. aureus*), neutrophils release NETs within 5–60 min independent of ROS produced by NADPH oxidase (NOX) (31) but depend on Toll-like receptor 2 (TLR2) and complement-mediated opsonization (32). This process starts with the separation of the inner and outer nuclear membranes and budding of vesicles. These vesicles containing DNA are delivered through the cytoplasm and released extracellularly (31) (**Figure 1B**). This rapid NETosis is also reported when neutrophils are stimulated by *Candida albicans* (33), *Leishmania* parasites (34), heparin (35), and LPS-stimulated platelets (36). Unlike in suicidal NETosis, neutrophils releasing NETs rapidly do not undergo cell lysis and remain functional, thus this process is termed as vital NETosis (37).

In addition to chromosomal DNA, mitochondrial DNA (mtDNA) has been reported as another source of NET DNA. Yousefi et al. demonstrated that neutrophils released mtDNA but not nuclear DNA in a ROS-dependent manner after priming of granulocyte/macrophage colony-stimulating factor (GM-CSF) and stimulation of LPS or complement factor 5a (C5a). In mtDNA, no nuclear proteins such as lamin B and nuclear matrix 45 (NP-45) were detected (38). Lood et al. demonstrated that ribonucleoprotein-containing immune complexes (RNP ICs), which are enriched in lupus, induced mobilization of mitochondria to the cell surface and release of mtDNA in a ROS-dependent manner (39). Van Dam et al. investigated the distinct pathways and characteristics of antineutrophil cytoplasmic antibody (ANCA)-associated vasculitis (AAV)-induced NETs and SLE-induced NETs. By quantifying the colocalization of TOMM20 and MitoSOX, the presence of mtDNA was confirmed in SLE-induced NETs but not in AAV-induced NETs (40).

Taken together, although pathways and mechanisms of NET formation are diverse and sophisticated upon different stimuli, they all come to an identical outcome: histone degradation, chromatin decondensation, and NETs released from neutrophils (41).

## The Von Willebrand Factor and ADAMTS13

### The Glycoprotein von Willebrand Factor

Von Willebrand factor, a glycoprotein released by endothelial cells or stimulated platelets, is essential in hemostasis (42). Mature VWF monomer contains three modules: D (D’-D3, D4), A (A1-A2-A3), and C (C1-C2-C3-C4-C5-C6-CK) (43). VWF monomers dimerize through the C-terminal cysteine knot (CK) domain in endoplasmic reticulum (ER). Dimers then assemble into multimers through D3 domain in Golgi (44). Interestingly, VWF multimers have been reported to self-associate forming branched structures under static and flow conditions (45–47). Notably, long VWF multimers, especially the UL-VWF exhibit prothrombotic activity (48). The activity of VWF is regulated by ADAMTS13 that specifically cleaves the peptide bond Tyr1605-Met1606 in A2 domain. VWF multimerization and ADAMTS13 proteolysis work together to balance the size of VWF multimers, ensuring the proper adhesion, activation, and aggregation of platelets. At sites of vascular injury, the circulating “coiled” VWF concatemers are immobilized through pairs of interactions, such as A3 domain binding to collagen I (49) and C domains binding to fibrin network (50). Then, the flowing blood stretches these immobilized VWF concatemers into an elongated conformation (51). The length of VWF multimers significantly affects their responses to shear. A lower threshold of shear rate is required to extend long VWF multimers (52). A recent research suggested that tension, but not shear stress, regulates the affinity of VWF for platelet glycoprotein Ibα (GPIbα) (53).

A1A2A3 tridomain is essential for VWF function, as it contains binding sites for GPIbα on platelets (A1), collagen in subendothelial matrix (A1 and A3), and the peptide bond cleaved by ADAMTS13 (A2). The function of A1A2A3 is modulated by shear stress. The A1-GPIbα bond exhibits force-enhanced characteristics, which could be explained by catch-bond (54) or flex-bond mechanism (55). Liu et al. applied molecular dynamics (MD) simulations to further examine how a mutation regulates the affinity of A1 to GPIbα (56). It was demonstrated that the destabilization of the N-terminal arm of gain-of-function (GoF) A1 mutant and the increased movement of the α2-helix could change the closed A1 conformation to a partially open state. The unfolding of the A2 domain is much easier than A1 and A3 domain, as the disulfide bond in A2 domain is located at the C-terminal end, which differs from the disulfide bond in A1 and A3 domain that links their C-terminal and N-terminal ends together (57). The unfolding forces for a single A2 domain range from 7 to 14 pN (58).

**Figure 2:**
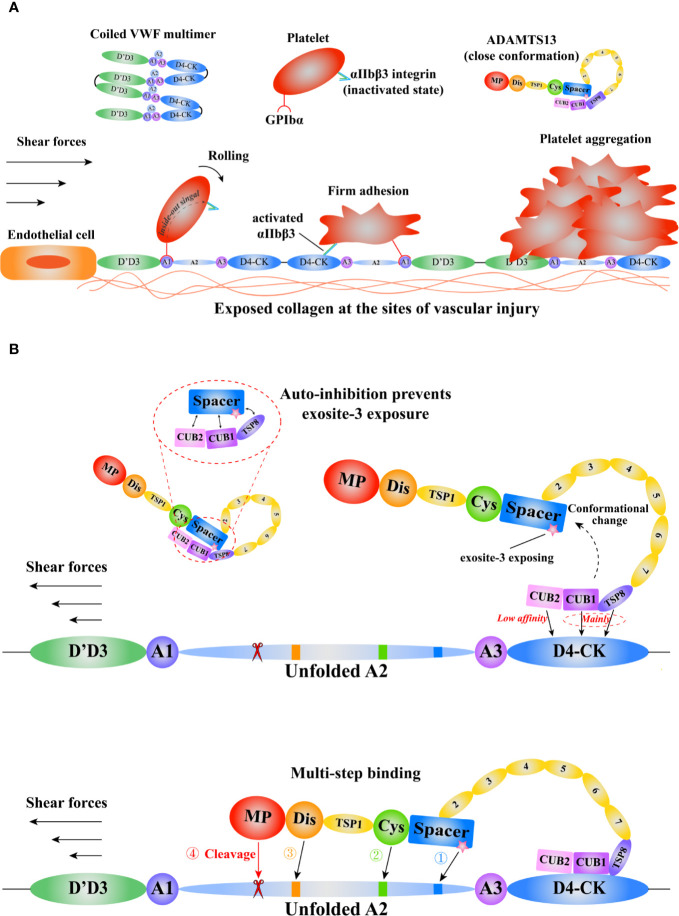
A schematic diagram of the mechanisms of VWF and ADAMTS13 in thrombus formation. Adapted from Petri et al. (64). **(A)** The function of VWF in thrombosis. The coiled VWF multimers tether to the exposed subendothelial collagen at sites of vascular injury. Then, the globular VWF is gradually stretched into an elongated state under tension. Subsequently, the platelet receptor GPIbα binds to the VWF A1domain to induce the rolling of platelet, and activate the integrin αIIbβ3 that interacts with D4-CK domains resulting in firm adhesion, promoting platelet aggregation. **(B)** The model of VWF-mediated conformational change of ADAMTS13 and subsequent multi-step binding process. The circulating ADAMTS13 in a closed conformation prevents the exposure of the exosite-3 in Spacer domain. This auto-inhibition of ADAMTS13 is relieved mainly through the interactions between ADAMTS13 TSP8-CUB1 domains and VWF D4-CK domains. Then, the high shear forces and flexible TSP type 1 repeats (T1-T8) assist the “open” ADAMTS13 to get close to the unfolded VWF A2 domain. After a multi-step binding, the MP domain recognizes and cleaves the scissile bond Y^1605^-M^1606^ in A2 domain. VWF, von Willebrand Factor; GPIbα, platelet glycoprotein Ibα; ADAMTS13, a disintegrin-like and metalloproteinase with thrombospondin type-1 motifs, member 13; TSP, thrombospondin; CUB, complement C1r/C1s, Uegf (epidermal growth factor–related sea urchin protein), and Bmp1 (bone morphogenetic protein 1); MP, metalloproteinase.

The function of A1A2A3 is also modulated by the interdomain interactions (59, 60). The binding of VWF A1 to GPIbα could be inhibited by its adjacent D’D3, A2, and A3 domains (8, 60, 61). The GPIbα binding site in A1 domain is exposed after the force-induced A2 domain unfolding (60). The half-life of A1-GPIbα bond is short, so it only can slow the rolling velocity of platelets (54). Subsequently, platelet receptor GPVI or β1 integrins (α2β1/α5β1) binds to respective ligands to form arrest adhesion (62). In addition, the integrin αIIbβ3 on platelet is activated through the “inside-out” and “outside-in” signaling, increasing the affinity to its ligands, such as VWF and fibrinogen, to contribute to platelet arrest adhesion and aggregation (63) (**Figure 2A**).

It has been demonstrated that VWF participates in leukocyte recruitment (65). The activated platelets on UL-VWF mediate leukocyte recruitment under static and flow conditions (65). Apart from its indirect role in leukocyte recruitment, VWF also directly regulates this process. The binding of D’D3 domain to P-selectin glycoprotein ligand 1 (PSGL-1) on leukocyte supports its rolling, and subsequently, VWF interacts with β_2_ integrin on leukocyte to promote its firm adhesion (66). In the presence of pathogens, VWF multimers also bind to NETs to form a network that can recruit both platelets and leukocytes and promote thrombosis (67). The size of such thrombi is efficiently controlled by ADAMTS13 and DNase I (68, 69). ADAMTS13 cleaves VWF multimers, while DNase I digests the DNA of NETs.

### The Metalloprotease ADAMTS13

ADAMTS13 specifically cleaves VWF to covert the large multimers into small ones. Severe deficiency in ADAMTS13 results in thrombotic thrombocytopenic purpura (TTP), a fatal disorder characterized by systemic microvascular thrombosis (70). ADAMTS13 is a multi-domain protein including a metalloprotease domain (M), a disintegrin-like domain (D), a thrombospondin Type-1 domain (T), a cysteine-rich domain (C), a spacer domain (S), followed by seven additional thrombospondin Type-1 repeats (T2–8) and two CUB (complement c1r/c1s, sea urchin epidermal growth factor, and bone morphogenetic protein) domains (71, 72). Evidence shows that various domains of ADAMTS13 bind to VWF (73, 74). Notably, the lack of spacer domain results in a dramatic reduction in its proteolytic activity, suggesting spacer domain is vital (75, 76). Although the crystal structure of full length ADAMS13 is not available to date, the crystal structure of its MDTCS provides critical insights into ADAMTS13 binding and cleavage (77, 78). Akiyama et al. firstly crystallized the DTCS structure in 2009 (79). Three exosites were reported on D, C, and S domain respectively. The cluster of four charged residues (Arg326, Glu327, His328, and Asp330) in the V-loop of D domain creates the exostie-1. De Groot et al. demonstrated that Arg349, Leu350, and Val352, close proximity to the cluster, interacted with unfolded VWF A2 (80), suggesting these residues function as a part of exosite-1. The V-loop in C domain creates another exosite (exosite-2). The cluster of His476/Ser477/Gln478 in the V-loop and the adjacent Arg488 play a pivotal role in VWF recognition. In addition, the U-loop and residues 494–496 flanking the V-loop may contribute to exosite-2. Exosite-3 comprises residues Tyr658, Arg659, Arg660, Tyr661, Tyr665, and two surrounding residues Arg568 and Phe692 in S domain. This exosite binds to the α6 helix of VWF A2 and is critical in substrate recognition and proteolysis (81). A “molecular zipper” model has been proposed to depict the multi-step process of ADAMTS13 binding to VWF (82). In this model, the distal T and CUB domains firstly bind to a constitutively exposed binding site in VWF D4CK, the discrete exosites, starting from exosite-3, then sequentially bind to different segments of unfolded A2, bringing the M domain and the VWF scissile bond into proximity. The S1 and S1’ subsites on the M domain engage with P1 Tyr1605 and P1’ Met1606 of VWF to allow the proteolysis to occur (**Figure 2B**). Recently, Petri et al. crystallized a Fab-MDTCS structure. The authors proposed that the M domain exhibited a latent conformation, and the binding of exosite-1 to VWF allosterically activated the M domain to facilitate proteolysis (77).

The distal T-CUB domains of ADAMTS13 interact with its S domain, resulting in a “closed” conformation with low activity, as demonstrated by the electron microscopy (83) and small-angle X-ray techniques (84). This autoinhibition is relieved by the binding of VWF D4CK or monoclonal antibodies to the distal domains or mutating five residues in S domain (R568K/F592Y/R660K/Y661F/Y665F), resulting in an “open” conformation and increasing the proteolytic activity ~2.5-fold (**Figure 2B**). Such mutated ADAMTS13 is termed as GoF-ADAMTS13 (85). By scanning WT- and GoF-ADAMTS13 molecules with atomic force microscopy (AFM), we proposed that ADAMTS13 might exist an additional “intermediate” conformation (86). However, further investigations are needed to reveal the physiological relevance of this conformation. South et al. reported that the GoF-ADAMTS13 and WT-MDTCS construct were able to cleave human fibrinogen and suggested that ADAMTS13 in the “closed” conformation restricted its specificity to prevent off-target proteolysis (83, 87). Three flexible linker segments between T2/T3, T4/T5, and T8/CUB1 contribute to ADAMTS13 conformation modulation. The removal of these three flexible segments increases the recognition of the cryptic epitope in M domain by the antibody 6A6, indicating that these three flexible segments modulate the binding of the distal domain to the proximal domain (88). South et al. proposed a model by integrating these findings to depict the conformational activation of ADAMTS13 by VWF (89).

Open ADAMTS13 conformation is found not only during acute acquired TTP (90), but also in patients in remission (91). Thus, open ADAMTS13 is proposed as a hallmark of acute acquired TTP and a novel biomarker to detect subclinical immune-mediated TTP in patients in remission as well. However, how the enhanced proteolytic activity of open ADAMTS13 is inhibited in acute acquired TTP patients remain elusive. It is possible that the exposure of cryptic epitopes in open ADAMTS13 may allow the binding of anti-spacer autoantibodies, thereby inhibiting ADAMTS13 function or inducing clearance of the resultant immune complexes. Designing new drugs to modulate ADAMTS13 conformation or prevent autoantibody binding might provide potential strategies for treating TTP.

### Mechanical Force Modulates the Proteolysis of VWF by ADAMTS13

It has been well established that blood flow mediates the recognition and cleavage processes of VWF by ADAMTS13 (73). As mentioned above, high shear stress unfolds VWF A2 domain to expose the scissile bond Tyr1605-Met1606 for ADAMTS13 cleavage, down-regulating the activity of VWF multimers and preventing excessive platelet aggregation. By the use of single-molecule techniques, we and other groups have demonstrated that A2 unfolding is the prerequisite for ADAMTS13 cleavage (92–94). Recently, we reported, for the first time, that the interactions between VWF and ADAMTS13 were modulated by mechanical forces, exhibiting multiple bond characteristics: slip bonds, catch bonds, biphasic bonds, and triphasic bonds (73). These dynamic bonds might be critical, as such interaction can be mechanically strengthened or weakened by the formation of various dynamic bonds between different binding sites in circulation. We also proposed a novel computer strategy combining the steered molecular dynamics simulation and flexible docking techniques to investigate the binding of S domain to α6 helix of VWF A2 with various extensions. The data demonstrated a biphasic extension-regulated binding of α6 helix to S domain, suggesting that S domain prefers to bind partially extended α6 helix (81).

## The NET-VWF Axis in Immunothrombosis Provides Novel Potential Therapeutic Strategies

### Mechanisms of NET-Induced Thrombosis

In recent years, NETs have been recognized as an important player in thrombosis (95, 96). NETs are found in thrombi from patients with venous or arterial thrombosis (96). The prothrombotic effect of NETs presents in triggering the coagulation pathway and platelet aggregation. NETs provide a scaffold to recruit red blood cells, platelets and leukocytes, as well as to bind plasma proteins, such as VWF and fibronectin (67). This scaffold also supports fibrin deposition by binding with fibrinogen. In addition, two primary components of NET structure, DNA and histone, are involved in the coagulation pathways. Cell-free DNA derived from NETs mediates thrombin generation in FXII- or FXI-dependent pathway but not in tissue factor (TF) related pathway in patients with sepsis (64). Digestion or precipitation of DNA networks markedly diminishes their procoagulant effects. Histones induce thrombin generation in platelet rich plasma (PRP) by activating platelets *via* TLR2 and TLR4, which is driven by poloP, an activator in clotting cascade secreted by activated platelets, in a FXII-independent manner (97). In addition, histones, especially H4, directly interact with platelets and activate αIIbβ3 integrin on platelet surface inducing subsequent fibrinogen mediated platelet aggregation (98). Histones also induce platelet micro-aggregation in an αIIbβ3-independent fibrinogen-dependent manner. Histones cause severe tissue damage and thrombocytopenia, and induce death in mice, which could be prevented by treatment with heparin (98). Interestingly, intact NETs exhibit weaker procoagulant effect than individual DNA and histone (99). The serine proteases NE and CG promote tissue factor- and factor XII-dependent coagulation and thrombus growth by counteracting endogenous anticoagulants (100).

TF is a key initiator of the extrinsic coagulation cascade. TF activates FVII to form TF/FVIIa complex that subsequently activates FIX and FX mediating ensuing thrombin and fibrin generation (101). Neutrophils immediately adhere to endothelial cells through the interaction between leukocyte function antigen-1 (LFA-1) and intercellular adhesion molecule-1 (ICAM-1) after laser-induced injury, and then express TF at the cell surface promoting fibrin generation and platelet aggregation *in vivo* (102), suggesting that neutrophils are a potential source of functional TF at the site of vascular injury. In addition, NETs also activate endothelial cells, inducing the elevated expression of vascular cell adhesion molecule-1 (VCAM-1), ICAM-1, and TF through interleukin-1α (IL-1α) and CG which could transform pro-IL-1α to mature IL-1α form (103). This NET-induced TF expression in endothelial cell surface accelerates plasma clotting *in vitro*. Elevated TF expression in neutrophils and the release of NETs decorated with TF have been found in patients with sepsis (104), AAV (105), ST-segment elevation acute myocardial infarction (STEMI) (106), SLE (107), and COVID-19 (108). In the courses of these diseases, neutrophils act as a pool of active TF. Neutrophilic TF is exposed by the formation of NETs triggered by activated platelets or stimuli in the pathological environment, which mediates localized thrombin generation and protease activated receptor-1 (PAR-1) signal-dependent platelet activation, promoting the formation of thrombus in these diseases. Neutrophils derived from healthy donors can form TF-bearing NETs stimulated by serum from these patients but not by PMA (103) or *Escherichia coli* (104), suggesting that elevated TF expression in neutrophils is mediated by special components in the pathological environment, such as cytokines in sepsis (104) and IgG in AAV (105).

The interaction between platelets and neutrophils plays a critical role in NET-induced thrombosis. Platelets interact with neutrophils to induce NETosis *via* the engagement of P-selectin/PSGL-1, the binding of neutrophil β_2_ integrins with GPIbα on platelet surface (109), and the release of soluble mediators, such as platelet factor 4 (PF4/CXCL4) (110) and high-mobility group box 1 (HMGB1) (111). HMGB1 enhances neutrophil recruitment and regulates NET formation (111, 112). Heterodimerization of CXCL4 and CCL5 enhances NET formation *via* integrin outside-in and G-protein-coupled receptor (GPCR) signaling (113). NETs in turn recruit and active more platelets. By increasing the expression of platelet P-selectin (114) or activating TLR2 and TLR4 (97), histones induce platelet activation. Histones also associate with platelets to induce platelet aggregation *via* the stimulation of calcium influx and recruitment of plasma adhesion proteins (98). A previous study by Sandra Grässle et al. in 2014 revealed that the isolated DNA of NETs directly interacts with platelet released VWF (14).

NETs are prothrombotic and play a critical role in thrombosis. Extensive reviews are provided elsewhere (96, 115). We will discuss the interactions among NETs, VWF, and ADAMTS13 in mediating immunothrombosis in diseases below.

### Mechanisms of NET-VWF Interaction and the Prothrombotic and Proinflammatory Effects

Pure DNA can directly bind to VWF A1 domain *via* electrostatic interactions (116), and this DNA-VWF interaction can be blocked by heparin (14). Notably, only activated VWF A1 domain can interact with isolated DNA and this interaction does not block the VWF cleavage by ADAMTS13. Preincubation of DNA significantly impairs the adhesion of platelets on the VWF coated surface under flow conditions, indicating that GPIbα binding site in VWF A1 domain is blocked by DNA. However, this blockage can be eliminated under physiological conditions, in which erythrocytes are present, in a hematocrit-dependent manner. *S. aureus*- or PMA-stimulated neutrophils adhere to VWF coated surface in microfluidic experiments, but this adhesion is completely abolished by the preincubation of heparin or the treatment of DNase I (14), suggesting that neutrophils can be recruited to vessel wall *via* the VWF-DNA interaction *in vitro*, apart from the reported binding of VWF/PSGL-1 and VWF/β_2_ integrin (66). A recent study reported that three arginine residues, Arg1392, Arg1395, and Arg1399, in helix 4 of the VWF A1 domain were the main binding sites for double-strand DNA (dsDNA). VWF-dsDNA interaction depended on ionic strength and shear stress-mediated VWF activation, but not nucleotide length and sequence (117). In contrast to abundant dsDNA adhered to the WT-VWF coated surface under high shear stress, almost no dsDNA adhesion was observed on the VWF R1399A mutant coated surface under the same shear stress, indicating that Arg1399 residue in VWF A1 is essential to the DNA-VWF interaction (117). Furthermore, purified soluble histone can also bind to VWF by electrostatic forces and this binding does not influence VWF adhesive activity (118). Neutrophil elastase, which contains positively charged side chain residues (119), is likely to bind to VWF *via* electrostatic interactions. Neutrophil elastases released from NETs still attached to the wall of liver vasculature after the treatment of DNase I in mice with methicillin-resistant *S. aureus* infection (15). Blocking VWF by antibody or cleaving VWF by ADAMTS13 did not affect neutrophil recruitment, but markedly prevented the accumulation of DNA fibers, decreased the amount of histone and elastase, and attenuated the tissue damage in liver sinusoid, supporting that the reduction of hepatic damage was due to the disruption of NET-VWF interaction (15). In a mouse model of hepatic ischemia-reperfusion injury, real-time intravital imaging demonstrated that leukocyte-vessel wall interactions in VWF knockout mice were significantly less than those in wild-type mice, supporting that VWF is a key mediator to recruit leukocytes to the sites of vascular damage (120). Less hepatic damage and better outcome were observed in VWF knockout mice or ADAMTS13 administered wild-type mice (120). In addition to endothelial cell-derived VWF, platelet-derived VWF is also associated with NETs. Carestia et al. reported that platelet-mediated NETs were significantly inhibited by blocking VWF released from activated platelets (110). They proposed that platelet-derived VWF might serve as a bridge that links activated platelets close to neutrophils, resulting in more platelet-mediated NET formation.

Previous studies have demonstrated that both VWF and NETs have proinflammatory effect (121, 122). In this point of view, the NET-VWF network, which attaches NET structure and recruits more leukocytes to stimulated endothelium, is likely not only to facilitate leukocyte infiltration into the surrounding tissue but also to amplify the proinflammatory effects of NETs. Thus, it is conceivable that VWF released from endothelial cells and platelets interacts with NETs to promote the progression of thrombosis and inflammation.

### NETs Mediating VWF and ADAMTS13 Activities May Form a Vicious Circle to Aggravate the Phenotype of Thrombotic Microangiopathies

NET contents mediate the activities of both VWF and ADAMTS13. Reduced ADAMTS13 activity concomitant with increased plasma VWF levels has been observed in thrombotic microangiopathies (TMAs) associated with systemic inflammation, such as in severe sepsis, sepsis-induced disseminated intravascular coagulation (DIC), malignancy, and autoimmune diseases (123). The mechanism is not fully understood, but activated neutrophils and NETosis may contribute to the deficiency of ADAMTS13. During systemic inflammation, activated neutrophils or NETosis release various cytokines, proteases, peptides, and ROS, such as hydrogen peroxide (H_2_O_2_) and hypochlorous acid (HOCl). Several inflammatory cytokines have been found to regulate the release and cleavage of UL-VWF under flow conditions (124). Interleukin 8, tumor necrosis factor α (TNF-α), and IL-6/IL-6 receptor complex stimulate the release of UL-VWF multimers, leading to VWF-platelet string formation on endothelial cell-cultured surface under flow. Notably, IL-6 inhibits the cleavage of UL-VWF by ADAMTS13 under flow but not in static conditions. In patients with sepsis-induced DIC, low molecular weight forms of ADAMTS13 are detected in plasma, indicating that ADAMTS13 deficiency may be due to the proteolysis of proteases in plasma apart from the impairment of biosynthesis (125). Neutrophil elastase and plasmin inactivate ADAMTS13 *via* proteolysis *in vitro* (125, 126) but not in the presence of human plasma under static conditions (127). Similarly, leukocyte proteases released from activated neutrophils or NETosis, including NE, PR3, CG, and MMP-9, cleave plasma-derived VWF at or near the ADAMTS13 cleavage site in a dose-dependent manner under shear stress *in vitro* (128). MPO catalyzes the generation of HOCl from H_2_O_2_ and Cl^–^ leading to oxidant tissue injury (129). Chen et al. reported that HOCl oxidized Met1606 at the ADAMTS13 cleavage site in VWF A2 domain, converting methionine to methionine sulfoxide, resulting in the oxidation of both VWF A2 peptide and plasma VWF multimers (130). These oxidized substrates were more difficult to be cleaved by ADAMTS13, indicating that this oxidative modification markedly impairs ADAMTS13 cleavage (130). Interestingly, HOCl generated by the MPO-H_2_O_2_-Cl^–^-system also oxidized methionine in ADAMTS13 and significantly inactivated ADAMTS13 to cleave VWF A2 peptide and plasma VWF multimers under denatured conditions (127). Further analysis by using LC-MS/MS showed that total seven methionine in MDTCS domains of ADAMTS13 were oxidized, among which Met249, Met331, and Met496 were critical for ADAMTS13 activity, and the extent of oxidation of each methionine was positively correlated with the reduction of ADAMTS13 activity. Notably, the oxidized ADAMTS13 could be further oxidized by HOCl with high concentration or activated neutrophils in the presence of 0.5% human plasma, resulting in marked activity loss. Even in the presence of 90% human plasma, >30% of Met249 and Met496 in ADAMTS13 were oxidized. These findings suggested that oxidation of ADAMTS13 by HOCl and subsequent proteolytic function impairment may occur in physiological conditions. Taken together, both VWF substrate and ADAMTS13 can be oxidized by MPO-H_2_O_2_-Cl^–^-system released from activated neutrophils or NETosis, leading to decreased ADAMTS13 activity and increased plasma VWF levels, eventually resulting in excessive platelet aggregation and occlusion at sites of vascular injury.

Human neutrophil peptides (HNPs), also known as α-defensins, play an important role in innate immune system (131). HNPs, which not only have proinflammatory effects but also enhance coagulation by activating platelets (132) and inhibiting fibrinolysis (133), have been observed abundantly in human atherosclerotic arteries (134) and increase in hyperlipidemia patients with coronary heart disease (135). HNPs released from activated neutrophils and NETosis also modulate the activities of both VWF and ADAMTS13. HNPs, mainly HNP1, HNP2, and HNP3, have antimicrobial properties and play an important role in innate immune response (136). Pillai et al. reported that HNPs dramatically inhibited the cleavage of FRET-VWF73 substrates and VWF multimers by ADAMTS13 *in vitro* (131). It might be due to HNPs and spacer domain of ADAMTS13 having the same RRY motif. HNPs competitively bound VWF A2 and blocked the interaction between ADAMTS13 and VWF, thereby impairing ADAMTS13 cleavage. Increased plasma levels of HNPs were observed in patients with acute TTP, implying the association between inflammation and ADAMTS13 deficiency in acute TTP (131). In patients with traumatic brain injury, high plasma levels of HNPs and VWF, concomitant with decreased plasma ADAMTS13 activity, associate with increased mortality (137). Surprisingly, HNPs also exhibit antithrombotic effects. HNP1 inhibits the adhesion and aggregation of murine platelets on the collagen-coated surface or on TNF-α activated endothelial cells under arterial shear stress in the absence of ADAMTS13 (138). Under arterial flow, VWF multimers are activated by high shear stress and expose the free cysteine thiols that facilitate VWF-VWF lateral association by forming new covalent disulfide bonds (139), leading to the formation of larger and thicker VWF multimer networks and the recruitment of more blood cells. HNP1 inhibits the formation of UL-VWF strings *via* the direct binding of its free cysteine thiols to free cysteine thiols in VWF (138).

During NET formation, PAD4, which converts positively charged arginine residues in histones to neutral citrulline residues (140), is essential for chromatin decondensation, and subsequently released with NETs. Recently, Sorvillo et al. demonstrated that PAD4 citrullinated plasma ADAMTS13 on specific arginine residues, resulting in a dramatic reduction of ADAMTS13 enzymatic activity and thus leading to increased VWF-platelet string formation in mesenteric venules of mice (141). PAD4 also decreased the time to vessel occlusion and markedly reduced thrombus embolization in murine mesenteric venules underwent ferric chloride-induced injury. Citrullinated ADAMTS13 was detected in plasma from healthy donors, septic patients and donors with other comorbidities, suggesting ADAMTS13 is citrullinated *in vivo* (141).

DNA-histone complexes released from NETosis or other forms of cell death are also associated with acute TMAs. In patients with acute TTP, elevated levels of DNA-histone complexes and MPO are detected compared to those in healthy donors, and these increased levels are inversely correlated with platelet counts, implying that DNA-histone complexes and MPO may contribute to thrombopenia (142). Zheng et al. successfully established novel *ADAMTS*13^–/–^ zebrafish lines that exhibited spontaneous but mild feature of TTP (143). They found that lysine-rich histone induced a more severe and persistent TTP phenotype and higher mortality rate in *ADAMTS*13^–/–^ than in wild-type zebrafish, indicating that histone might trigger and aggravate TTP in individuals with severe ADAMTS13 deficiency. Increased plasma levels of VWF antigen were also observed in *ADAMTS*13^–/–^ zebrafish after treatment with histone. Both spontaneous and histone-induced TTP phenotypes were effectively prevented in *ADAMTS*13^–/–^ *via* VWF gene knock-out, suggesting that VWF might be a potential therapeutic target in inflammation-induced acute TTP phenotype (143).

**Figure 3:**
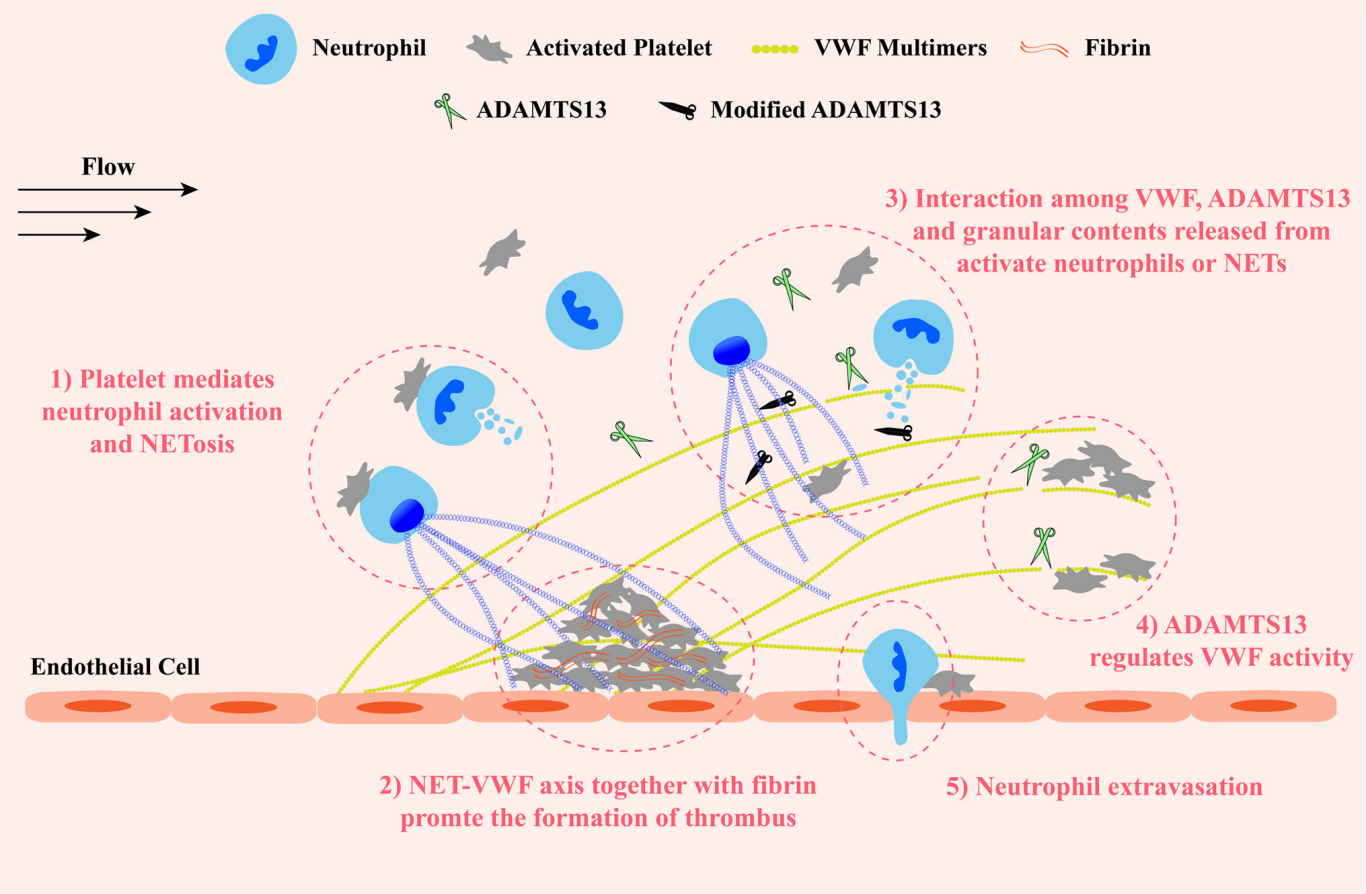
A schematic diagram of the VWF-NET axis in thrombus formation and tissue damage at sites of vascular injury. Upon damage, VWF multimers are released from endothelial cells and capture circulating platelets to injury sites. VWF multimers recruit blood cells including neutrophils and erythrocytes. ADAMTS13 cleaves excessive VWF multimers to regulate their activity preventing the growth of thrombus. 1) The interaction among neutrophils, platelets, and VWF forms a vicious cycle to promote the development of thrombosis and inflammation: platelets mediate neutrophil activation and NETosis, then NETs and granular proteins released from activated neutrophils are retained to the vessel wall by directly interacting with VWF and exert prothrombotic and proinflammatory effects, inducing the release of VWF multimers and promoting platelet adhesion in turn. 2) Both NETs and VWF modify fibrin networks enhancing their procoagulant activity and the resistance of fibrinolysis providing insight into potential therapeutic targets of rt-PA resistance thrombi. 3) Granular components released from activated neutrophils and NETosis not only inactivate ADAMTS13 *via* chemical modification or competitive combination but also cleave VWF. 4) ADAMTS13 binds and cleaves VWF multimers to prevent excessive platelet aggregation. 5) Neutrophils extravagates into the intima with the help of VWF-platelet complex. VWF, von Willebrand Factor; ADAMTS13, a disintegrin-like and metalloproteinase with thrombospondin type-1 motifs, member 13.

Taken together, contents released from activated neutrophils and NETosis, on the one hand, inhibit ADAMTS13 activity by oxidation, citrullination, proteolysis or competitively binding to VWF A2 substrate, resulting in the increased concentration of VWF antigen, promoting the formation of UL-VWF multimers and their prothrombotic effects at sites of vascular injury. On the other hand, like ADAMTS13, several proteases cleave VWF, exhibiting their antithrombotic effects. However, whether these antithrombotic effects work *in vivo* is uncertain. These findings shed light on the relationship between inflammation and ADAMTS13 deficiency in TMAs. The interaction between NETs and VWF retains abundant NET components and activated neutrophils on damaged vessel wall. More granulocyte contents are in close proximity to VWF and VWF-bound ADAMTS13, and exert their prothrombotic effects to promote the development of thrombosis. This NET-VWF interaction may lead a vicious cycle: NET component-mediated deficiency of ADAMTS13 promotes the formation of UL-VWF multimers which in turn recruit and attach more activated neutrophils and NET components to vessel wall, aggravating the symptom of inflammation and thrombosis (**Figure 3**). Therefore, targeting NET-VWF axis by the administration of recombinant human ADAMTS13 and/or DNase I, maybe a potential therapeutic strategy for TMAs and other systemic inflammatory diseases.

### NET-VWF Axis Is a Potential Therapeutic Target for Acute Ischemic Stroke

NETs and VWF play a critical role in the pathophysiology of acute ischemic stroke (AIS) (144, 145). AIS is caused by occlusion and stenosis of internal carotid artery (ICA) or vertebral artery, which obstruct cerebral blood flow and eventually result in brain damage. AIS causes massive death worldwide and only two treatments are approved by the FDA to date (1): mechanical removal of thrombus *via* endovascular thrombectomy, and (2) pharmacological thrombolysis by using t-PA that promotes degradation of fibrin in thrombus by activating endogenous plasminogen (146). However, endovascular treatment is not always available and t-PA therapy is limited by the narrow therapeutic time window (4.5 h after stroke onset). It has been proposed that NETs contained in thrombi may be one of the reasons for treatment failure. Abundant neutrophils and NETs are observed in almost all thrombi retrieved from patients with ischemic stroke (147). Removing NETs by DNase I or impeding NET formation by Cl-amidine, a PAD4 inhibitor, significantly inhibited arterial thrombosis in ischemic mouse brain and ameliorated stroke outcome, such as decreasing the infarct volume and maintaining the basal blood flow level (146). *In vitro* experiments showed that addition of extracellular DNA and histones to fibrin increased the thickness, stiffness and stability of the fibrin network, making the clot harder to be dissolved by t-PA, but the treatment with both DNase I and t-PA showed significant clot lysis (148). These findings were further supported by Ducroux et al. (149). They found that NET content in thrombi from AIS patients was positively associated with endovascular procedure length and device number of passes, and targeting NETs by DNase I accelerated t-PA-induced thrombolysis in *ex vivo* assay. These results suggested that NETs alter the mechanical properties of thrombus, increasing the resistance of thrombus to endovascular treatment and recombinant t-PA (rt-PA) therapy (149). Recent clinical analysis also supported that high numbers of NETs enhanced the compactness and stability of stroke thrombi, making the vessels harder to be recanalized and resulting in worse clinical outcome (150).

It has been reported that VWF is incorporated into fibrin network *via* covalently crosslinking by factor X IIIa (151) or in a thrombin-dependent manner (50). Thrombi from ischemic stroke patients contain on average 20.3 ± 10.1% of VWF (152), and VWF (especially endothelial cell-derived VWF) is proved to mediate ischemic stroke by promoting postischemic thrombo-inflammation in a brain ischemic/reperfusion injury model (153). Infusion of recombination human ADAMTS13 markedly dissolved the t-PA resistant thrombi in a dose-dependent manner, resulting in reduced cerebral infarct sizes in a mice model with *FeCl*_3_-induced middle cerebral artery occlusion (152). AIS patients with low ADAMTS13 activity may have poor early neurological improvement after rt-PA therapy, implying that ADAMTS13 favors rt-PA to perform its thrombolytic effect (154). A recent study further analyzed the components of the thrombi from ischemic stroke patients at the molecular and cellular levels (12). In sections of stroke thrombi, leukocytes and extracellular DNA mainly presented in platelet-rich regions and the boundary areas between platelet-rich and red blood cell-rich regions (12). Interestingly, in addition to abundant leukocytes and extracellular DNA, the interaction between dense thin fibrin and VWF was observed and platelets were filled within these fibrin-VWF structures in platelet-rich regions (12). VWF serves as a mediator to bring NETs and fibrin into close proximity, facilitating NETs to modify fibrin structure and to perform its procoagulant effect. rt-PA is the only thrombolytic drug approved by the FDA for ischemic stroke, but it works only for less than half of patients (12, 152). The reason for the rt-PA resistance is not fully understood, but it is believed that excessive NETs presented in thrombus are the critical factor. Targeting the NET-VWF axis seem to be a promising and effective intravenous thrombolysis therapy than existing rt-PA treatments in rt-PA-resistant thrombi, as Staessens et al. have discussed (12).

The reason for excessive NET formation in AIS thrombus is unclear, however, it is reasonable to speculate that platelets play an important role in uncontrolled NETosis and the following rt-PA resistance in stroke thrombus. Denorme et al. had discussed that VWF-GPIbα is a thrombo-inflammatory axis in ischemic stroke (155). Endothelial cell-bound VWF activated by hydrodynamic force mediates initial platelet adhesion *via* the binding of its A1 domain to platelet GPIbα to allow subsequent platelet activation and aggregation. In contrast to its direct binding to endothelial cell *via* P-selectin/PSGL-1 interaction under venous low shear stress, leukocytes are recruited to endothelial surface under arterial high shear stress by the ideal substrate, VWF-platelet complex (65). This complex can also alter vascular permeability and thus promote leukocyte extravasation (156, 157). Breaking the interaction between VWF and GPIbα markedly reduced brain injury and improve functional outcome in murine stroke model (155). Similarly, NET formation mediated by platelet TLR4 promoted the growth of arterial thrombus in an ischemic stroke mouse model (146). DNase I responded thrombi from patients with AIS had denser platelet distribution than those DNase I nonresponded ones. Thrombi with higher platelet density showed more weight loss after DNase I treatment. These results implied that the amount of NETs is associated with the density of platelets in stroke thrombi. Moreover, a recent study reported that interactions between NETs and activated platelets played an important role in the hypercoagulability of stroke patients with ICA occlusion (158). Plasma from carotid lesion site in stroke patients not only induced platelet activation in a thrombin-dependent manner, but also exposed phosphatidylserine (PS, a critical catalyst in the coagulation cascade) on the surfaces of platelets, platelet-derived microparticles (PMPs) and neutrophils. Activated platelets mediated the formation of NETs decorated with PS. These PS-bearing NETs were observed in the carotid thrombus and provided a platform to bind PMPs and coagulation factors, including prothrombin, fibrinogen, and Factor X, and thus promoted thrombin and fibrin formation (158).

Taken together, both NETs and VWF can not only recruit platelets and leukocytes, but also promote clot coagulation and leukocytes infiltration. It is tempting to speculate that NET-VWF interaction provides a larger and more stable network structure for the recruitment of blood cells and the binding of coagulation factors at sites of vascular injury under flow, facilitating the interactions among the components, such as platelets/leukocytes/NETs and fibrin/VWF/NETs, and thus promoting the development of thrombosis and inflammation, aggravating vessel and organ damage (**Figure 3**). Therefore, targeting the NET-VWF axis by DNase I and/or ADAMTS13 is a potential therapeutic strategy for AIS.

### NET-VWF Axis Is a Potential Therapeutic Target in COVID-19

Recently, COVID-19, which is caused by severe acute respiratory syndrome coronavirus 2 (SARS-CoV-2) (159), is a global health care challenge, with rapid spread and high mortality (160). COVID-19 is characterized by acute respiratory distress syndrome (ARDS), in which, acute respiratory failure, endothelial injury, immunothrombosis, and imbalance between coagulation and inflammation have been reported (161, 162). Zuo et al. reported that high levels of NETs were found in sera from patients with COVID-19, and these sera could trigger the formation of NETs from neutrophils collected from healthy volunteers *in vitro* (160). A further study proved that SARS-CoV-2 could directly trigger NET formation *via* an ACE2-serine protease TMPRSS2 dependent pathway and induce lung epithelial cell death *in vitro* (163). Nicolai et al. demonstrated that in COVID-19 patients, inflammatory microvascular thrombi containing NETs were present in lung, kidney, and heart (164). Middleton et al. also demonstrated that elevated levels of PF4 and RANTES, which were demonstrated to trigger NETosis, were detected in plasma from COVID-19 patients and COVID-19 plasma-induced NET formation was inhibited by neonatal NET-Inhibitory Factor (nNIF), indicating that NETs contributed to immunothrombosis in COVID-19 patients (165). In addition, thrombocytopenia, hyperactive coagulation, pulmonary damage, acute cardiac and kidney injuries were observed in patients with severe COVID-19 (166–168). Barnes et al. observed extensive neutrophil infiltration in pulmonary capillaries from the autopsy specimen of a COVID-19 patient and proposed that therapies targeting NETs might alleviate the severity of COVID-19 (167). NET formation, which results in the production of inflammatory cytokines, might contribute to cytokine storm and worse outcome of COVID-19 (167, 169). COVID-19 and NET-associated diseases share common manifestations, it thus is reasonable to propose that NETs may be a novel therapeutic target for COVID-19 (167). Dornase alfa, the recombinant human DNase I, is FDA-approved for cystic fibrosis treatment (170). It is also used off-label as mucolytic in other diseases, such as ARDS, and may be beneficial in the context of COVID-19 (171). Nine such clinical trials are registered (*NCT04432987*, *NCT04387786*, *NCT04409925*, *NCT04359654*, *NCT04445285*, *NCT04402944*, *NCT04355364*, *NCT04402970*, and *NCT04459325*), including two trials are in phase 3 (*NCT04355364* and *NCT04402970*) and one trial is completed (*NCT04459325*).

Along with NETs, VWF and ADAMTS13 are also reported to be involved in COVID-19. Ladikou et al. observed high levels of VWF and coagulation factor VIII that released from injured endothelial cells could contribute to hypercoagulability and the elevated rate of venous thromboembolism (VTE) in COVID-19 patients, suggesting that VWF could be used for the hierarchy of endothelial damage and thrombotic risk (172). By assessing the markers of endothelial cell and platelet activation, Goshua et al. demonstrated that mortality of COVID-19 patients was correlated with VWF antigen and soluble thrombomodulin (173). In addition, the reduction of ADAMST13 was observed in COVID-19 patients and proposed to use to predict mortality (174). However, normal ADAMTS13 activity was also observed in COVID-19 inpatients (175).

Although the involvement of NETs and VWF in endothelial damage and COVID-19 has been elucidated, no direct evidence yet demonstrates their interactions contributing to COVID-19 progression. Further studies are necessary to shed new light on the interactions and the mechanisms of NET-VWF in COVID-19, and provide the potential therapeutic strategies.

## Conclusions

The field of NETosis is in an exciting phase. Most of the literature mainly focuses on which stimuli can induce NET formation and which proteins can inhibit the process. The well-defined singling pathway that is distinct from other cell death pathways remains elusive. In addition, the initiation of NETosis is still an open question: what determines NETosis to occur in response to the stimulation that also triggers phagocytosis and degranulation. Recently, Xie et al. identified eight neutrophil populations by profiling >25,000 differentiating and mature mouse neutrophils using single-cell RNA sequencing (176). Further investigations are needed to reveal whether different neutrophil populations possess different capabilities to generate NETs in response to stimuli. New findings would allow us to better define the physiological significance of NETosis.

Over the past decade, abundant elegant animal and human studies have demonstrated that NETs contribute to thrombus formation and propagation in arterial, venous, and cancer-associated thrombosis. The interactions between NETs and VWF form a larger and thicker net-work accelerating the immunothrombosis in diseases. Combinational usage of DNase I and ADAMTS13 is therefore a potential therapeutic strategy. Several studies have investigated the bindings of VWF A1 to NET DNA and histone. Further studies are needed to provide more insights into the basis of the molecular structure of these bindings. The new findings might facilitate the designs of new drugs selectively against the NET-VWF binding, which might be crucial for the patients with acquired TTP. Notably, the conformation and function of VWF are regulated by the blood flow. Whether and how the blood flow alters the binding between NETs and VWF is an interesting question and less explored. Apart from the NET-VWF interaction, we extensively review the direct or indirect inactivation of ADAMTS13 by granulocyte contents released from activated neutrophils or NETosis. It is interesting to investigate whether the modifications of ADAMTS13 by NETs modulate its conformation and thus the enzymatic activity.

The interplay among NET, VWF and ADAMTS13 might form a vicious cycle, resulting in reduced ADAMTS13 activity and subsequently elevated plasma levels of VWF, which is positively correlated with severity and mortality in TMAs, AIS, and COVID-19. Targeting the NET-VWF axis may pave a new road to therapeutic strategies for immunothrombosis in diseases.

## Author Contributions

JL and JY designed the paper. JL, JY, ZW, QL, JH, TH, and WL wrote the paper. All authors contributed to the article and approved the submitted version.

## Funding

This work was supported by National Natural Science Foundation of China Grants (31771012 to JL), the High-level Hospital Construction Project of Guangdong Provincial People’s Hospital (KJ012020057 to JL).

## Conflict of Interest

The authors declare that the research was conducted in the absence of any commercial or financial relationships that could be construed as a potential conflict of interest.

## References

[1] Papayannopoulos V Neutrophil extracellular traps in immunity and disease. Nat Rev Immunol (2018) 18:134–47. 10.1038/nri.2017.105 28990587

[2] Brinkmann V Reichard U Goosmann C Fauler B Uhlemann Y Weiss DS Neutrophil Extracellular Traps Kill Bacteria. Science (2004) 303:1532–5. 10.1126/science.1092385 15001782

[3] Frangou E Vassilopoulos D Boletis J Boumpas DT An emerging role of neutrophils and NETosis in chronic inflammation and fibrosis in systemic lupus erythematosus (SLE) and ANCA-associated vasculitides (AAV): Implications for the pathogenesis and treatment. Autoimmun Rev (2019) 18:751–60. 10.1016/j.autrev.2019.06.011 31181324

[4] Denning NL Aziz M Gurien SD Wang P Damps and nets in sepsis. Front Immunol (2019) 10:2536. 10.3389/fimmu.2019.02536 31736963 PMC6831555

[5] Albrengues J Shields MA Ng D Park CG Ambrico A Poindexter ME Neutrophil extracellular traps produced during inflammation awaken dormant cancer cells in mice. Science (2018) 361:eaao4227. 10.1126/science.aao4227 30262472 PMC6777850

[6] Döring Y Soehnlein O Weber C Neutrophil extracellular traps in atherosclerosis and atherothrombosis. Circ Res (2017) 120:736–43. 10.1161/CIRCRESAHA.116.309692 28209798

[7] Moschonas IC Tselepis AD The pathway of neutrophil extracellular traps towards atherosclerosis and thrombosis. Atherosclerosis (2019) 288:9–16. 10.1016/j.atherosclerosis.2019.06.919 31280097

[8] Löf A Müller JP Brehm MA A biophysical view on von Willebrand factor activation. J Cell Physiol (2018) 233:799–810. 10.1002/jcp.25887 28256724

[9] Zhang C Kelkar A Neelamegham S Von Willebrand factor self-association is regulated by the shear-dependent unfolding of the A2 domain. Blood Adv (2019) 3:957–68. 10.1182/bloodadvances.2018030122 PMC6457215 30936056

[10] South K Lane DA ADAMTS-13 and von Willebrand factor: a dynamic duo. J Thromb Haemost (2018) 16:6–18. 10.1111/jth.13898 29108103 PMC5814862

[11] Brill A Fuchs TA Savchenko AS Thomas GM Martinod K de Meyer SF Neutrophil extracellular traps promote deep vein thrombosis in mice. J Thromb Haemost (2012) 10:136–44. 10.1111/j.1538-7836.2011.04544.x PMC3319651 22044575

[12] Staessens S Denorme F François O Desender L Dewaele T Vanacker P Structural analysis of ischemic stroke thrombi: histological indications for therapy resistance. Haematologica (2020) 105:498–507. 10.3324/haematol.2019.219881 31048352 PMC7012484

[13] Thålin C Hisada Y Lundström S Mackman N Wallén H Neutrophil Extracellular Traps. Arterioscler Thromb Vasc Biol (2019) 39:1724–38. 10.1161/ATVBAHA.119.312463 PMC6703916 31315434

[14] Grässle S Huck V Pappelbaum KI Gorzelanny C Aponte-Santamaría C Baldauf C Von willebrand factor directly interacts with DNA from neutrophil extracellular traps. Arterioscler Thromb Vasc Biol (2014) 34:1382–9. 10.1161/ATVBAHA.113.303016 24790143

[15] Kolaczkowska E Jenne CN Surewaard BGJ Thanabalasuriar A Lee WY Sanz MJ Molecular mechanisms of NET formation and degradation revealed by intravital imaging in the liver vasculature. Nat Commun (2015) 6:1–13. 10.1038/ncomms7673 PMC4389265 25809117

[16] Delgado-Rizo V Martínez-Guzmán MA Iñiguez-Gutierrez L García-Orozco A Alvarado-Navarro A Fafutis-Morris M Neutrophil extracellular traps and its implications in inflammation: An overview. Front Immunol (2017) 8:81. 10.3389/fimmu.2017.00081 28220120 PMC5292617

[17] Cao W Pham HP Williams LA McDaniel J Siniard RC Lorenz RG Human neutrophil peptides and complement factor Bb in pathogenesis of acquired thrombotic thrombocytopenic purpura. Haematologica (2016) 101:1319–26. 10.3324/haematol.2016.149021 PMC5394873 27662014

[18] Urban CF Ermert D Schmid M Abu-Abed U Goosmann C Nacken W Neutrophil extracellular traps contain calprotectin, a cytosolic protein complex involved in host defense against Candida albicans. PLoS Pathog (2009) 5:e1000639. 10.1371/journal.ppat.1000639 19876394 PMC2763347

[19] Petretto A Bruschi M Pratesi F Croia C Candiano G Ghiggeri G Neutrophil extracellular traps (NET) induced by different stimuli: A comparative proteomic analysis. PLoS One (2019) 14:e0218946. 10.1371/journal.pone.0218946 31283757 PMC6613696

[20] Pires RH Felix SB Delcea M The architecture of neutrophil extracellular traps investigated by atomic force microscopy. Nanoscale (2016) 8:14193–202. 10.1039/c6nr03416k 27387552

[21] Thiama HR Wong SL Qiu R Kittisopikul M Vahabikashi A Goldman AE NETosis proceeds by cytoskeleton and endomembrane disassembly and PAD4-mediated chromatin decondensation and nuclear envelope rupture. Proc Natl Acad Sci U S A (2020) 117:7326–37. 10.1073/pnas.1909546117 PMC7132277 32170015

[22] Neubert E Meyer D Rocca F Günay G Kwaczala-Tessmann A Grandke J Chromatin swelling drives neutrophil extracellular trap release. Nat Commun (2018) 9:1–13. 10.1038/s41467-018-06263-5 30218080 PMC6138659

[23] Neubert E Meyer D Kruss S Erpenbeck L The power from within - understanding the driving forces of neutrophil extracellular trap formation. J Cell Sci (2020) 133:jcs241075. 10.1242/jcs.241075 32156720

[24] Metzler KD Goosmann C Lubojemska A Zychlinsky A Papayannopoulos V Myeloperoxidase-containing complex regulates neutrophil elastase release and actin dynamics during NETosis. Cell Rep (2014) 8:883–96. 10.1016/j.celrep.2014.06.044 PMC4471680 25066128

[25] Papayannopoulos V Metzler KD Hakkim A Zychlinsky A Neutrophil elastase and myeloperoxidase regulate the formation of neutrophil extracellular traps. J Cell Biol (2010) 191:677–91. 10.1083/jcb.201006052 PMC3003309 20974816

[26] Sorvillo N Cherpokova D Martinod K Wagner DD Extracellular DNA net-works with dire consequences for health. Circ Res (2019) 125:470–88. 10.1161/CIRCRESAHA.119.314581 PMC6746252 31518165

[27] Wu SY Weng CL Jheng MJ Kan HW Hsieh ST Liu FT Candida albicans triggers NADPH oxidaseindependent neutrophil extracellular traps through dectin-2. PLoS Pathog (2019) 15:e1008096. 10.1371/journal.ppat.1008096 31693704 PMC6834254

[28] de Souza CN Breda LCD Khan MA de Almeida SR Câmara NOS Sweezey N Alkaline pH promotes NADPH oxidase-independent neutrophil extracellular trap formation: A matter of mitochondrial reactive oxygen species generation and citrullination and cleavage of histone. Front Immunol (2018) 8:1849. 10.3389/fimmu.2017.01849 29375550 PMC5767187

[29] Rohrbach AS Slade DJ Thompson PR Mowen KA Activation of PAD4 in NET formation. Front Immunol (2012) 3:360. 10.3389/fimmu.2012.00360 23264775 PMC3525017

[30] Silva JC Rodrigues NC Thompson-Souza GA Muniz V de S Neves JS Figueiredo RT Mac-1 triggers neutrophil DNA extracellular trap formation to Aspergillus fumigatus independently of PAD4 histone citrullination. J Leukoc Biol (2020) 107:69–83. 10.1002/JLB.4A0119-009RR 31478251

[31] Pilsczek FH Salina D Poon KKH Fahey C Yipp BG Sibley CD A Novel Mechanism of Rapid Nuclear Neutrophil Extracellular Trap Formation in Response to Staphylococcus aureus. J Immunol (2010) 185:7413–25. 10.4049/jimmunol.1000675 21098229

[32] Yipp BG Petri B Salina D Jenne CN Scott BNV Zbytnuik LD Infection-induced NETosis is a dynamic process involving neutrophil multitasking in vivo. Nat Med (2012) 18:1386–93. 10.1038/nm.2847 PMC4529131 22922410

[33] Byrd AS O’Brien XM Johnson CM Lavigne LM Reichner JS An Extracellular Matrix–Based Mechanism of Rapid Neutrophil Extracellular Trap Formation in Response to Candida albicans. J Immunol (2013) 190:4136–48. 10.4049/jimmunol.1202671 PMC3622194 23509360

[34] Rochael NC Guimarães-Costa AB Nascimento MTC Desouza-Vieira TS Oliveira MP Garciae Souza LF Classical ROS-dependent and early/rapid ROS-independent release of Neutrophil Extracellular Traps triggered by Leishmania parasites. Sci Rep (2015) 5:1–11. 10.1038/srep18302 PMC4682131 26673780

[35] Lelliott PM Momota M Shibahara T Lee MSJ Smith NI Ishii KJ Heparin induces neutrophil elastase-dependent vital and lytic NET formation. Int Immunol (2020) 32:359–68. 10.1093/intimm/dxz084 31879779

[36] Clark SR Ma AC Tavener SA McDonald B Goodarzi Z Kelly MM Platelet TLR4 activates neutrophil extracellular traps to ensnare bacteria in septic blood. Nat Med (2007) 13:463–9. 10.1038/nm1565 17384648

[37] Yipp BG Kubes P NETosis: How vital is it? Blood (2013) 122:2784–94. 10.1182/blood-2013-04-457671 24009232

[38] Yousefi S Mihalache C Kozlowski E Schmid I Simon HU Viable neutrophils release mitochondrial DNA to form neutrophil extracellular traps. Cell Death Differ (2009) 16:1438–44. 10.1038/cdd.2009.96 19609275

[39] Lood C Blanco LP Purmalek MM Carmona-Rivera C De Ravin SS Smith CK Neutrophil extracellular traps enriched in oxidized mitochondrial DNA are interferogenic and contribute to lupus-like disease. Nat Med (2016) 22:146–53. 10.1038/nm.4027 PMC4742415 26779811

[40] van Dam LS Kraaij T Kamerling SWA Bakker JA Scherer UH Rabelink TJ Intrinsically Distinct Role of Neutrophil Extracellular Trap Formation in Antineutrophil Cytoplasmic Antibody–Associated Vasculitis Compared to Systemic Lupus Erythematosus. Arthritis Rheumatol (2019) 71:2047–58. 10.1002/art.41047 PMC7384043 31313503

[41] Kenny EF Herzig A Krüger R Muth A Mondal S Thompson PR Diverse stimuli engage different neutrophil extracellular trap pathways. Elife (2017) 6:101–6. 10.7554/eLife.24437 PMC5496738 28574339

[42] Brehm MA Von Willebrand factor processing. Hamostaseologie (2017) 37:59–72. 10.5482/HAMO-16-06-0018 28139814

[43] Zhou YF Eng ET Zhu J Lu C Walz T Springer TA Sequence and structure relationships within von Willebrand factor. Blood (2012) 120:449–58. 10.1182/blood-2012-01-405134 PMC3398765 22490677

[44] Springer TA Von Willebrand factor, Jedi knight of the bloodstream. Blood (2014) 124:1412–25. 10.1182/blood-2014-05-378638 PMC4148764 24928861

[45] Li Y Choi H Zhou Z Nolasco L Pownall HJ Voorberg J Covalent regulation of ULVWF string formation and elongation on endothelial cells under flow conditions. J Thromb Haemost (2008) 6:1135–43. 10.1111/j.1538-7836.2008.02991.x PMC2532495 18433456

[46] Ulrichts H Vanhoorelbeke K Girma JP Lenting PJ Vauterin S Deckmyn H The von Willebrand factor self-association is modulated by a multiple domain interaction. J Thromb Haemost (2005) 3:552–61. 10.1111/j.1538-7836.2005.01209.x 15748246

[47] Savage B Sixma JJ Ruggeri ZM Functional self-association of von Willebrand factor during platelet adhesion under flow. Proc Natl Acad Sci U S A (2002) 99:425–30. 10.1073/pnas.012459599 PMC117576 11756664

[48] Furlan M Von Willebrand factor: Molecular size and functional activity. Ann Hematol (1996) 72:341–8. 10.1007/s002770050184 8767102

[49] Romijn RA Westein E Bouma B Schiphorst ME Sixma JJ Lenting PJ Mapping the collagen-binding site in the von Willebrand factor-A3 domain. J Biol Chem (2003) 278:15035–9. 10.1074/jbc.M208977200 12582178

[50] Miszta A Pelkmans L Lindhout T Krishnamoorthy G De Groot PG Hemker CH Thrombin-dependent Incorporation of von Willebrand factor into a Fibrin network. J Biol Chem (2014) 289:35979–86. 10.1074/jbc.M114.591677 PMC4276865 25381443

[51] Siedlecki CA Lestini BJ Kottke-Marchant K Eppell SJ Wilson DL Marchant RE Shear-dependent changes in the three-dimensional structure of human von Willebrand factor. Blood (1996) 88:2939–50. 10.1182/blood.v88.8.2939.bloodjournal8882939 8874190

[52] Wang Y Morabito M Zhang XF Webb E Oztekin A Cheng X Shear-Induced Extensional Response Behaviors of Tethered von Willebrand Factor. Biophys J (2019) 116:2092–102. 10.1016/j.bpj.2019.04.025 PMC6554656 31103230

[53] Fu H Jiang Y Yang D Scheiflinger F Wong WP Springer TA Flow-induced elongation of von Willebrand factor precedes tension-dependent activation. Nat Commun (2017) 8:324. 10.1038/s41467-017-00230-2 28831047 PMC5567343

[54] Yago T Lou J Wu T Yang J Miner JJ Coburn L Platelet glycoprotein Ibα forms catch bonds with human WT vWF but not with type 2B von Willebrand disease vWF. J Clin Invest (2008) 118:3195–207. 10.1172/JCI35754 PMC2518822 18725999

[55] Kim J Zhang CZ Zhang X Springer TA A mechanically stabilized receptor-ligand flex-bond important in the vasculature. Nature (2010) 466:992–5. 10.1038/nature09295 PMC4117310 20725043

[56] Liu G Fang Y Wu J A mechanism for localized dynamics-driven affinity regulation of the binding of von willebrand factor to platelet glycoprotein Ibα. J Biol Chem (2013) 288:26658–67. 10.1074/jbc.M113.453803 PMC3772212 23902764

[57] Butera D Passam F Ju L Cook KM Woon H Aponte-Santamaría C Autoregulation of von Willebrand factor function by a disulfide bond switch. Sci Adv (2018) 4:eaaq1477. 10.1126/sciadv.aaq1477 29507883 PMC5834005

[58] Zhang X Halvorsen K Zhang CZ Wong WP Springer TA Mechanoenzymatic cleavage of the ultralarge vascular protein von willebrand factor. Science (2009) 324:1330–4. 10.1126/science.1170905 PMC2753189 19498171

[59] Ulrichts H Udvardy M Lenting PJ Pareyn I Vandeputte N Vanhoorelbeke K Shielding of the A1 domain by the D′D3 domains of von Willebrand factor modulates its interaction with platelet glycoprotein Ib-IX-V. J Biol Chem (2006) 281:4699–707. 10.1074/jbc.M513314200 16373331

[60] Martin C Morales LD Cruz MA Purified A2 domain of von Willebrand factor binds to the active conformation of von Willebrand factor and blocks the interaction with platelet glycoprotein Ibα. J Thromb Haemost (2007) 5:1363–70. 10.1111/j.1538-7836.2007.02536.x 17389010

[61] Obert B Houllier A Meyer D Girma JP Conformational changes in the A3 domain of von Willebrand factor modulate the interaction of the A1 domain with platelet glycoprotein Ib. Blood (1999) 93:1959–68. 10.1182/blood.v93.6.1959.406k01_1959_1968 10068669

[62] Scharf RE Platelet Signaling in Primary Haemostasis and Arterial Thrombus Formation ∗: Part 1. Hamostaseologie (2018) 38:203–10. 10.1055/s-0038-1675144 30352470

[63] Yuan H Deng N Zhang S Cao Y Wang Q Liu X The unfolded von Willebrand factor response in bloodstream: The self-association perspective. J Hematol Oncol (2012) 5:65. 10.1186/1756-8722-5-65 23067373 PMC3488313

[64] Gould TJ Vu TT Swystun LL Dwivedi DJ Mai SHC Weitz JI Neutrophil extracellular traps promote thrombin generation through platelet-dependent and platelet-independent mechanisms. Arterioscler Thromb Vasc Biol (2014) 34:1977–84. 10.1161/ATVBAHA.114.304114 25012129

[65] Bernardo A Ball C Nolasco L Choi H Moake JL Dong JF Platelets adhered to endothelial cell-bound ultra-large von Willebrand factor strings support leukocyte tethering and rolling under high shear stress. J Thromb Haemost (2005) 3:562–70. 10.1111/j.1538-7836.2005.01122.x 15748247

[66] Pendu R Terraube V Christophe OD Gahmberg CG De Groot PG Lenting PJ P-selectin glycoprotein ligand 1 and β2-integrins cooperate in the adhesion of leukocytes to von Willebrand factor. Blood (2006) 108:3746–52. 10.1182/blood-2006-03-010322 16926295

[67] Fuchs TA Brill A Duerschmied D Schatzberg D Monestier M Myers DD Extracellular DNA traps promote thrombosis. Proc Natl Acad Sci U S A (2010) 107:15880–5. 10.1073/pnas.1005743107 PMC2936604 20798043

[68] Lancellotti S Basso M De Cristofaro R Proteolytic processing of Von Willebrand Factor by Adamts13 and Leukocyte Proteases. Mediterr J Hematol Infect Dis (2013) 5:e2013058. 10.4084/mjhid.2013.058 24106608 PMC3787661

[69] Weber C Jenke A Chobanova V Yazdanyar M Chekhoeva A Eghbalzadeh K Targeting of cell-free DNA by DNase I diminishes endothelial dysfunction and inflammation in a rat model of cardiopulmonary bypass. Sci Rep (2019) 9:19249. 10.1038/s41598-019-55863-8 31848423 PMC6917735

[70] Scully M Cataland SR Peyvandi F Coppo P Knöl P Kremer Hovinga JA Caplacizumab treatment for acquired thrombotic thrombocytopenic purpura. N Engl J Med (2019) 380:335–46. 10.1056/NEJMoa1806311 30625070

[71] Soejima K Mimura N Hirashima M Maeda H Hamamoto T Nakagaki T A novel human metalloprotease synthesized in the liver and secreted into the blood: Possibly, the von Willebrand factor-cleaving protease? J Biochem (2001) 130:475–80. 10.1093/oxfordjournals.jbchem.a003009 11574066

[72] Dong JF Structural and functional correlation of ADAMTS13. Curr Opin Hematol (2007) 14:270–6. 10.1097/MOH.0b013e3280d35820 17414218

[73] Li Z Lin J Sulchek T Cruz MA Wu J Zhu C Domain-specific mechanical modulation of VWF–ADAMTS13 interaction. Mol Biol Cell (2019) 30:1920–9. 10.1091/mbc.E19-01-0021 PMC6727775 31067148

[74] Zanardelli S Chion ACK Groot E Lenting PJ Mckinnon TAJ Laffan MA A novel binding site for ADAMTS13 constitutively exposed on the surface of globular VWF. Blood (2009) 114:2819–29. 10.1182/blood-2009-05-224915 PMC3402367 19587373

[75] Tao Z Wang Y Choi H Bernardo A Nishio K Sadler JE Cleavage of ultralarge multimers of von Willebrand factor by C-terminal-truncated mutants of ADAMTS-13 under flow. Blood (2005) 106:141–3. 10.1182/blood-2004-11-4188 PMC1895119 15774619

[76] Papers JBC Doi M Zheng X Nishio K Majerus EM Sadler JE Cleavage of von Willebrand Factor Requires the Spacer Domain of the Metalloprotease ADAMTS13 *. J Biol Chem (2003) 278:30136–41. 10.1074/jbc.M305331200 PMC11033693 12791682

[77] Petri A Kim HJ Xu Y de Groot R Li C Vandenbulcke A Crystal structure and substrate-induced activation of ADAMTS13. Nat Commun (2019) 10:1–16. 10.1038/s41467-019-11474-5 31439947 PMC6706451

[78] Schelpe AS Petri A Roose E Pareyn I Deckmyn H De Meyer SF Antibodies that conformationally activate ADAMTS13 allosterically enhance metalloprotease domain function. Blood Adv (2020) 4:1072–80. 10.1182/bloodadvances.2019001375 PMC7094026 32196558

[79] Akiyama M Takeda S Kokame K Takagi J Miyata T Crystal structures of the noncatalytic domains of ADAMTS13 reveal multiple discontinuous exosites for von Willebrand factor. Proc Natl Acad Sci U S A (2009) 106:19274–9. 10.1073/pnas.0909755106 PMC2780749 19880749

[80] De Groot R Bardhan A Ramroop N Lane DA Crawley JTB Essential role of the disintegrin-like domain in ADAMTS13 function. Blood (2009) 113:5609–16. 10.1182/blood-2008-11-187914 19234142

[81] Fang X Lin J Fang Y Wu J Prediction of spacer-α6 complex: A novel insight into binding of ADAMTS13 with A2 domain of von Willebrand factor under forces. Sci Rep (2018) 8:1–12. 10.1038/s41598-018-24212-6 29636514 PMC5893608

[82] Crawley JTB De Groot R Xiang Y Luken BM Lane DA Unraveling the scissile bond: How ADAMTS13 recognizes and cleaves von Willebrand factor. Blood (2011) 118:3212–21. 10.1182/blood-2011-02-306597 PMC3179391 21715306

[83] South K Luken BM Crawley JTB Phillips R Thomas M Collins RF Conformational activation of ADAMTS13. Proc Natl Acad Sci (2014) 111:18578–83. 10.1073/pnas.1411979112 PMC4284544 25512499

[84] Muia J Zhu J Gupta G Haberichter SL Friedman KD Feys HB Allosteric activation of ADAMTS13 by von Willebrand factor. Proc Natl Acad Sci U S A (2014) 111:18584–9. 10.1073/pnas.1413282112 PMC4284596 25512528

[85] Jian C Xiao J Gong L Skipwith CG Jin S Kwaan HC Gain-of-function ADAMTS13 variants that are resistant to autoantibodies against ADAMTS13 in patients with acquired thrombotic thrombocytopenic purpura. Blood (2012) 119:3836–44. 10.1182/blood-2011-12-399501.The PMC3335387 22289888

[86] Yu S Liu W Fang J Shi X Wu J Fang Y AFM Imaging Reveals Multiple Conformational States of ADAMTS13. J Biol Eng (2019) 13:1–11. 10.1186/s13036-018-0102-y 30679946 PMC6343300

[87] South K Freitas MO Lane DA Conformational quiescence of ADAMTS-13 prevents proteolytic promiscuity. J Thromb Haemost (2016) 14:2011–22. 10.1111/jth.13445 PMC5111603 27514025

[88] Deforche L Roose E Vandenbulcke A Vandeputte N Feys HB Springer TA Linker regions and flexibility around the metalloprotease domain account for conformational activation of ADAMTS-13. J Thromb Haemost (2015) 13:2063–75. 10.1111/jth.13149 PMC4778570 26391536

[89] South K Freitas MO Lane DA A model for the conformational activation of the structurally quiescent metalloprotease ADAMTS13 by von willebrand factor. J Biol Chem (2017) 292:5760–9. 10.1074/jbc.M117.776732 PMC5392571 28209710

[90] Roose E Schelpe AS Joly BS Peetermans M Verhamme P Voorberg J An open conformation of ADAMTS-13 is a hallmark of acute acquired thrombotic thrombocytopenic purpura. J Thromb Haemost (2018) 16:378–88. 10.1111/jth.13922 29222940

[91] Roose E Schelpe AS Tellier E Sinkovits G Joly BS Dekimpe C Open ADAMTS13, induced by antibodies, is a biomarker for subclinical immune-mediated thrombotic thrombocytopenic purpura. Blood (2020) 136:353–61. 10.1182/blood.2019004221 32356859

[92] Wu T Lin J Cruz MA Dong JF Zhu C Force-induced cleavage of single VWFA1A2A3 tridomains by ADAMTS-13. Blood (2010) 115:370–8. 10.1182/blood-2009-03-210369 PMC2808159 19897584

[93] Ying J Ling Y Westfield LA Sadler JE Shao JY Unfolding the a2 domain of von willebrand factor with the optical trap. Biophys J (2010) 98:1685–93. 10.1016/j.bpj.2009.12.4324 PMC2856187 20409490

[94] Löf A Walker PU Sedlak SM Gruber S Obser T Brehm MA SUP: Multiplexed protein force spectroscopy reveals equilibrium protein folding dynamics and the low-force response of von Willebrand factor. Proc Natl Acad Sci U S A (2019) 116:18798–807. 10.1073/pnas.1901794116 PMC6754583 31462494

[95] Martinod K Wagner DD Thrombosis: Tangled up in NETs. Blood (2014) 123:2768–76. 10.1182/blood-2013-10-463646 PMC4007606 24366358

[96] Laridan E Martinod K De Meyer SF Neutrophil Extracellular Traps in Arterial and Venous Thrombosis. Semin Thromb Hemost (2019) 45:86–93. 10.1055/s-0038-1677040 30634198

[97] Semeraro F Ammollo CT Morrissey JH Dale GL Friese P Esmon NL Extracellular histones promote thrombin generation through platelet-dependent mechanisms: Involvement of platelet TLR2 and TLR4. Blood (2011) 118:1952–61. 10.1182/blood-2011-03-343061 PMC3158722 21673343

[98] Fuchs TA Bhandari AA Wagner DD Histones induce rapid and profound thrombocytopenia in mice. Blood (2011) 118:3708–14. 10.1182/blood-2011-01-332676 PMC3186342 21700775

[99] Noubouossie DF Whelihan MF Bin Y Sparkenbaugh E Pawlinski R Monroe DM In vitro activation of coagulation by human neutrophil DNA and histone proteins but not neutrophil extracellular traps. Blood (2017) 129:1021–9. 10.1182/blood-2016-06-722298 PMC5324715 27919911

[100] Massberg S Grahl L Von Bruehl ML Manukyan D Pfeiler S Goosmann C Reciprocal coupling of coagulation and innate immunity via neutrophil serine proteases. Nat Med (2010) 16:887–96. 10.1038/nm.2184 20676107

[101] Grover SP Mackman N Tissue Factor: An Essential Mediator of Hemostasis and Trigger of Thrombosis. Arterioscler Thromb Vasc Biol (2018) 38:709–25. 10.1161/ATVBAHA.117.309846 29437578

[102] Darbousset R Thomas GM Mezouar S Frère C Bonier R Mackman N Tissue factor-positive neutrophils bind to injured endothelial wall and initiate thrombus formation. Blood (2012) 120:2133–43. 10.1182/blood-2012-06-437772 22837532

[103] Folco EJ Mawson TL Vromman A Bernardes-Souza B Franck G Persson O Neutrophil extracellular traps induce endothelial cell activation and tissue factor production through interleukin-1α and cathepsin G. Arterioscler Thromb Vasc Biol (2018) 38:1901–12. 10.1161/ATVBAHA.118.311150 PMC6202190 29976772

[104] Kambas K Mitroulis I Apostolidou E Girod A Chrysanthopoulou A Pneumatikos I Autophagy Mediates the Delivery of Thrombogenic Tissue Factor to Neutrophil Extracellular Traps in Human Sepsis. PLoS One (2012) 7:1–14. 10.1371/journal.pone.0045427 PMC3446899 23029002

[105] Kambas K Chrysanthopoulou A Vassilopoulos D Apostolidou E Skendros P Girod A Tissue factor expression in neutrophil extracellular traps and neutrophil derived microparticles in antineutrophil cytoplasmic antibody associated vasculitis may promote thromboinflammation and the thrombophilic state associated with the disease. Ann Rheum Dis (2014) 73:1854–63. 10.1136/annrheumdis-2013-203430 23873874

[106] Stakos DA Kambas K Konstantinidis T Mitroulis I Apostolidou E Arelaki S Expression of functional tissue factor by neutrophil extracellular traps in culprit artery of acute myocardial infarction. Eur Heart J (2015) 36:1405–14. 10.1093/eurheartj/ehv007 PMC4458286 25660055

[107] Frangou E Chrysanthopoulou A Mitsios A Kambas K Arelaki S Angelidou I REDD1/autophagy pathway promotes thromboinflammation and fibrosis in human systemic lupus erythematosus (SLE) through NETs decorated with tissue factor (TF) and interleukin-17A (IL-17A). Ann Rheum Dis (2019) 78:238–48. 10.1136/annrheumdis-2018-213181 PMC6352428 30563869

[108] Skendros P Mitsios A Chrysanthopoulou A Mastellos DC Metallidis S Rafailidis P Complement and tissue factor-enriched neutrophil extracellular traps are key drivers in COVID-19 immunothrombosis. J Clin Invest (2020) 6:141374. 10.1172/jci141374 PMC7598040 32759504

[109] Lisman T Platelet–neutrophil interactions as drivers of inflammatory and thrombotic disease. Cell Tissue Res (2018) 371:567–76. 10.1007/s00441-017-2727-4 PMC5820397 29178039

[110] Carestia A Kaufman T Rivadeneyra L Landoni VI Pozner RG Negrotto S Mediators and molecular pathways involved in the regulation of neutrophil extracellular trap formation mediated by activated platelets. J Leukoc Biol (2016) 99:153–62. 10.1189/jlb.3a0415-161r 26320263

[111] Maugeri N Campana L Gavina M Covino C De Metrio M Panciroli C Activated platelets present high mobility group box 1 to neutrophils, inducing autophagy and promoting the extrusion of neutrophil extracellular traps. J Thromb Haemost (2014) 12:2074–88. 10.1111/jth.12710 25163512

[112] Dyer MR Chen Q Haldeman S Yazdani H Hoffman R Loughran P Deep vein thrombosis in mice is regulated by platelet HMGB1 through release of neutrophil-extracellular traps and DNA. Sci Rep (2018) 8:1–7. 10.1038/s41598-018-20479-x 29391442 PMC5794752

[113] Rossaint J Herter JM Van Aken H Napirei M Döring Y Weber C Synchronized integrin engagement and chemokine activation is crucial in neutrophil extracellular trap-mediated sterile inflammation. Blood (2014) 123:2573–84. 10.1182/blood-2013-07-516484 24335230

[114] André P P-selectin in haemostasis. Br J Haematol (2004) 126:298–306. 10.1111/j.1365-2141.2004.05032.x 15257701

[115] Noubouossie DF Reeves BN Strahl BD Key NS Neutrophils: Back in the thrombosis spotlight. Blood (2019) 133:2186–97. 10.1182/blood-2018-10-862243 PMC7218731 30898858

[116] Huang RH Fremont DH Diener JL Schaub RG Sadler JE A Structural Explanation for the Antithrombotic Activity of ARC1172, a DNA Aptamer that Binds von Willebrand Factor Domain A1. Structure (2009) 17:1476–84. 10.1016/j.str.2009.09.011 PMC3845234 19913482

[117] Sandoval-Pérez A Berger RML Garaizar A Farr SE Brehm MA König G DNA binds to a specific site of the adhesive blood-protein von Willebrand factor guided by electrostatic interactions. Nucleic Acids Res (2020) 48:7333–44. 10.1093/nar/gkaa466 PMC7367192 32496552

[118] Ward CM Tetaz TJ Andrews RK Berndt MC Binding of the von Willebrand factor A1 domain to histone. Thromb Res (1997) 86:469–77. 10.1016/S0049-3848(97)00096-0 9219327

[119] Edwards JV Howley PS Human neutrophil elastase and collagenase sequestration with phosphorylated cotton wound dressings. J BioMed Mater Res A (2007) 79:963–73. 10.1002/jbm.a 17477392

[120] Urisono Y Sakata A Matsui H Kasuda S Ono S Yoshimoto K Von Willebrand Factor Aggravates Hepatic Ischemia-Reperfusion Injury by Promoting Neutrophil Recruitment in Mice. Thromb Haemost (2018) 118:700–8. 10.1055/s-0038-1636529 29618155

[121] Gragnano F Sperlongano S Golia E Natale F Bianchi R Crisci M The Role of von Willebrand Factor in Vascular Inflammation: From Pathogenesis to Targeted Therapy. Mediators Inflammation (2017) 2017:5620314. 10.1155/2017/5620314 PMC5467347 28634421

[122] Honda M Kubes P Neutrophils and neutrophil extracellular traps in the liver and gastrointestinal system. Nat Rev Gastroenterol Hepatol (2018) 15:206–21. 10.1038/nrgastro.2017.183 29382950

[123] Farkas P Csuka D Mikes B Sinkovits G Réti M Németh E Complement activation, inflammation and relative ADAMTS13 deficiency in secondary thrombotic microangiopathies. Immunobiology (2017) 222:119–27. 10.1016/j.imbio.2016.10.014 27771173

[124] Bernardo A Ball C Nolasco L Moake JF Dong JF Effects of inflammatory cytokines on the release and cleavage of the endothelial cell-derived ultralarge von Willebrand-factor multimers under flow. Blood (2004) 104:100–6. 10.1182/blood-2004-01-0107 15026315

[125] Ono T Mimuro J Madoiwa S Soejima K Kashiwakura Y Ishiwata A Severe secondary deficiency of von Willebrand factor-cleaving protease (ADAMTS13) in patients with sepsis-induced disseminated intravascular coagulation: Its correlation with development of renal failure. Blood (2006) 107:528–34. 10.1182/blood-2005-03-1087 16189276

[126] Crawley JTB Lam JK Rance JB Mollica LR O’Donnell JS Lane DA Proteolytic inactivation of ADAMTS13 by thrombin and plasmin. Blood (2005) 105:1085–93. 10.1182/blood-2004-03-1101 15388580

[127] Wang Y Chen J Ling M López JA Chung DW Fu X Hypochlorous acid generated by neutrophils inactivates ADAMTS13: An oxidative mechanism for regulating ADAMTS13 proteolytic activity during inflammation. J Biol Chem (2015) 290:1422–31. 10.1074/jbc.M114.599084 PMC4340389 25422322

[128] Raife TJ Cao W Atkinson BS Bedell B Montgomery RR Lentz SR Leukocyte proteases cleave von Willebrand factor at or near the ADAMTS13 cleavage site. Blood (2009) 114:1666–74. 10.1182/blood-2009-01-195461 PMC2731642 19541819

[129] Seymour J Klebanoff. Myeloperox: friend and foe. J Leukoc Biol (2005) 77:598–625. 10.1189/jlb.1204697.1 15689384

[130] Chen J Fu X Wang Y Ling M Mcmullen B Kulman J Oxidative modification of von Willebrand factor by neutrophil oxidants inhibits its cleavage by ADAMTS13. Blood (2009) 115:1–3. 10.1182/blood-2009-03-213967.An PMC2810979 19812385

[131] Pillai VG Bao J Zander CB McDaniel JK Chetty PS Seeholzer SH Human neutrophil peptides inhibit cleavage of von Willebrand factor by ADAMTS13: A potential link of inflammation to TTP. Blood (2016) 128:110–9. 10.1182/blood-2015-12-688747 PMC4937355 27207796

[132] Quinn K Henriques M Parker T Slutsky AS Zhang H Human neutrophil peptides: A novel potential mediator of inflammatory cardiovascular diseases. Am J Physiol Heart Circ Physiol (2008) 295:H1817–24. 10.1152/ajpheart.00472.2008 PMC4896811 18805897

[133] Higazi AAR Ganz T Kariko K Cines DB Defensin modulates tissue-type plasminogen activator and plasminogen binding to fibrin and endothelial cells. J Biol Chem (1996) 271:17650–5. 10.1074/jbc.271.30.17650 8663495

[134] Barnathan ES Raghunath PN Tomaszewski JE Ganz T Cines DB Al-Roof Higazi A Immunohistochemical localization of defensin in human coronary vessels. Am J Pathol (1997) 150:1009–20. PMC1857878 9060838

[135] Maneerat Y Prasongsukarn K Benjathummarak S Dechkhajorn W Chaisri U Increased alpha-defensin expression is associated with risk of coronary heart disease: A feasible predictive inflammatory biomarker of coronary heart disease in hyperlipidemia patients. Lipids Health Dis (2016) 15:1–12. 10.1186/s12944-016-0285-5 27430968 PMC4949746

[136] Lehrer RI Lu W α-Defensins in human innate immunity. Immunol Rev (2012) 245:84–112. 10.1111/j.1600-065X.2011.01082.x 22168415

[137] Kumar MA Cao W Pham HP Raju D Nawalinski K Maloney-Wilensky E Relative Deficiency of Plasma A Disintegrin and Metalloprotease with Thrombospondin Type 1 Repeats 13 Activity and Elevation of Human Neutrophil Peptides in Patients with Traumatic Brain Injury. J Neurotrauma (2019) 36:222–9. 10.1089/neu.2018.5696 PMC6338577 29848170

[138] McDaniel JK Abdelgawwad MS Hargett A Renfrow MB Bdeir K Cao W Human neutrophil peptide-1 inhibits thrombus formation under arterial flow via its terminal free cysteine thiols. J Thromb Haemost (2019) 17:596–606. 10.1111/jth.14407 30741476 PMC6443439

[139] Schneider SW Nuschele S Wixforth A Gorzelanny C Alexander-Katz A Netz RR Shear-induced unfolding triggers adhesion of von Willebrand factor fibers. Proc Natl Acad Sci U S A (2007) 104:7899–903. 10.1073/pnas.0608422104 PMC1876544 17470810

[140] Wong SL Wagner DD Peptidylarginine deiminase 4: A nuclear button triggering neutrophil extracellular traps in inflammatory diseases and aging. FASEB J (2018) 32:6358–70. 10.1096/fj.201800691R PMC6219837 29924943

[141] Sorvillo N Mizurini DM Coxon C Martinod K Tilvawala R Cherpokova D Plasma peptidylarginine deiminase IV promotes VWF-platelet string formation and accelerates thrombosis after vessel injury. Circ Res (2019) 125:507–19. 10.1161/CIRCRESAHA.118.314571 PMC6697196 31248335

[142] Fuchs TA Kremer Hovinga JA Schatzberg D Wagner DD Lämmle B Circulating DNA and myeloperoxidase indicate disease activity in patients with thrombotic microangiopathies. Blood (2012) 120:1157–64. 10.1182/blood-2012-02-412197 PMC3418712 22611154

[143] Zheng L Abdelgawwad MS Zhang D Xu L Wei S Cao W Histone-induced thrombotic thrombocytopenic purpura in adamts13-/- zebrafish depends on von Willebrand factor. Haematologica (2020) 105:1107–19. 10.3324/haematol.2019.237396 PMC7109750 31753928

[144] Vallés J Lago A Santos MT Latorre AM Tembl JI Salom JB Neutrophil extracellular traps are increased in patients with acute ischemic stroke: Prognostic significance. Thromb Haemost (2017) 117:1919–29. 10.1160/TH17-02-0130 28837206

[145] Buchtele N Schwameis M Gilbert JC Schörgenhofer C Jilma B Targeting von Willebrand Factor in Ischaemic Stroke: Focus on Clinical Evidence. Thromb Haemost (2018) 118:959–78. 10.1055/s-0038-1648251 PMC6193403 29847840

[146] Peña-Martínez C Durán-Laforet V García-Culebras A Ostos F Hernández-Jiménez M Bravo-Ferrer I Pharmacological Modulation of Neutrophil Extracellular Traps Reverses Thrombotic Stroke tPA (Tissue-Type Plasminogen Activator) Resistance. Stroke (2019) 50:3228–37. 10.1161/STROKEAHA.119.026848 31526124

[147] Laridan E Denorme F Desender L François O Andersson T Deckmyn H Neutrophil extracellular traps in ischemic stroke thrombi. Ann Neurol (2017) 82:223–32. 10.1002/ana.24993 28696508

[148] Longstaff C Varjú I Sótonyi P Szabó L Krumrey M Hoell A Mechanical stability and fibrinolytic resistance of clots containing fibrin, DNA, and histones. J Biol Chem (2013) 288:6946–56. 10.1074/jbc.M112.404301 PMC3591605 23293023

[149] Ducroux C Di Meglio L Loyau S Delbosc S Boisseau W Deschildre C Thrombus neutrophil extracellular traps content impair tPA-induced thrombolysis in acute ischemic stroke. Stroke (2018) 49:754–7. 10.1161/STROKEAHA.117.019896 29438080

[150] Novotny J Oberdieck P Titova A Pelisek J Chandraratne S Nicol P Thrombus NET content is associated with clinical outcome in stroke and myocardial infarction. Neurology (2020) 94:e2346–60. 10.1212/WNL.0000000000009532 32434865

[151] Bockenstedt P Mcdonagh J Covalent Crosslinking of vwf to fibrin. Blood (1986) 68:95–101. 10.1182/blood.V68.1.95.bloodjournal68195 2872929

[152] Denorme F Langhauser F Desender L Vandenbulcke A Rottensteiner H Plaimauer B ADAMTS13-mediated thrombolysis of t-PA-resistant occlusions in ischemic stroke in mice. Blood (2016) 127:2337–45. 10.1182/blood-2015-08-662650 26929275

[153] Dhanesha N Prakash P Doddapattar P Khanna I Pollpeter MJ Nayak MK Endothelial cell-derived von willebrand factor is the major determinant that mediates von willebrand factor-dependent acute Ischemic Stroke by promoting postischemic thrombo-inflammation. Arterioscler Thromb Vasc Biol (2016) 36:1829–37. 10.1161/ATVBAHA.116.307660 PMC5001895 27444201

[154] Putzer AS Worthmann H Grosse GM Goetz F Martens-Lobenhoffer J Dirks M ADAMTS13 activity is associated with early neurological improvement in acute ischemic stroke patients treated with intravenous thrombolysis. J Thromb Thrombolysis (2020) 49:67–74. 10.1007/s11239-019-01941-7 31482326

[155] Denorme F Vanhoorelbeke K De Meyer SF von Willebrand Factor and Platelet Glycoprotein Ib: A Thromboinflammatory Axis in Stroke. Front Immunol (2019) 10:2884. 10.3389/fimmu.2019.02884 31921147 PMC6928043

[156] Petri B Broermann A Li H Khandoga AG Zarbock A Krombach F Von Willebrand factor promotes leukocyte extravasation. Blood (2010) 116:4712–9. 10.1182/blood-2010-03-276311 20716766

[157] Gros A Ollivier V Ho-Tin-Noé B Platelets in inflammation: Regulation of leukocyte activities and vascular repair. Front Immunol (2015) 6:678. 10.3389/fimmu.2014.00678 PMC4285099 25610439

[158] Zhou P Li T Jin J Liu Y Li B Sun Q Interactions between neutrophil extracellular traps and activated platelets enhance procoagulant activity in acute stroke patients with ICA occlusion. EbioMedicine (2020) 53:102671. 10.1016/j.ebiom.2020.102671 32114386 PMC7047181

[159] Gorbalenya AE Baker SC Baric RS de Groot RJ Drosten C Gulyaeva AA The species Severe acute respiratory syndrome-related coronavirus: classifying 2019-nCoV and naming it SARS-CoV-2. Nat Microbiol (2020) 5:536–44. 10.1038/s41564-020-0695-z PMC7095448 32123347

[160] Zuo Y Yalavarthi S Shi H Gockman K Zuo M Madison JA Neutrophil extracellular traps in COVID-19. JCI Insight (2020) 5:e138999. 10.1172/jci.insight.138999 PMC7308057 32329756

[161] Potey PMD Rossi AG Lucas CD Dorward DA Neutrophils in the initiation and resolution of acute pulmonary inflammation: understanding biological function and therapeutic potential. J Pathol (2019) 247:672–85. 10.1002/path.5221 PMC6492013 30570146

[162] Frantzeskaki F Armaganidis A Orfanos SE Immunothrombosis in Acute Respiratory Distress Syndrome: Cross Talks between Inflammation and Coagulation. Respiration (2017) 93:212–25. 10.1159/000453002 27997925

[163] Veras FP Pontelli M Silva C Toller-Kawahisa J de Lima M Nascimento D SARS-CoV-2 triggered neutrophil extracellular traps (NETs) mediate COVID-19 pathology. J Exp Med (2020) 217:e20201129. 10.1101/2020.06.08.20125823 32926098 PMC7488868

[164] Nicolai L Leunig A Brambs S Kaiser R Weinberger T Weigand M Immunothrombotic Dysregulation in COVID-19 Pneumonia Is Associated With Respiratory Failure and Coagulopathy. Circulation (2020) 142:1176–89. 10.1161/CIRCULATIONAHA.120.048488 PMC7497892 32755393

[165] Middleton EA He X-Y Denorme F Campbell RA Ng D Salvatore SP Neutrophil Extracellular Traps (NETs) Contribute to Immunothrombosis in COVID-19 Acute Respiratory Distress Syndrome. Blood (2020) 136:1169–79. 10.1182/blood.2020007008 PMC7472714 32597954

[166] Zhang Y Xiao M Zhang S Xia P Cao W Jiang W Coagulopathy and antiphospholipid antibodies in patients with covid-19. N Engl J Med (2020) 382:E38. 10.1056/NEJMc2007575 32268022 PMC7161262

[167] Barnes BJ Adrover JM Baxter-Stoltzfus A Borczuk A Cools-Lartigue J Crawford JM Targeting potential drivers of COVID-19: Neutrophil extracellular traps. J Exp Med (2020) 217:1–7. 10.1084/jem.20200652 PMC7161085 32302401

[168] Bonow RO Fonarow GC O’Gara PT Yancy CW Association of Coronavirus Disease 2019 (COVID-19) with Myocardial Injury and Mortality. JAMA Cardiol (2020) 5:751–3. 10.1001/jamacardio.2020.1105 32219362

[169] Tomar B Anders HJ Desai J Mulay SR Neutrophils and Neutrophil Extracellular Traps Drive Necroinflammation in COVID-19. Cells (2020) 9:1–8. 10.3390/cells9061383 PMC7348784 32498376

[170] Weber AG Chau AS Egeblad M Barnes BJ Janowitz T Nebulized in-line endotracheal dornase alfa and albuterol administered to mechanically ventilated COVID-19 patients: a case series. Mol Med (2020) 26:91. 10.1186/s10020-020-00215-w 32993479 PMC7522910

[171] Radermecker C Detrembleur N Guiot J Cavalier E Henket M D’Emal C Neutrophil extracellular traps infiltrate the lung airway, interstitial, and vascular compartments in severe COVID-19. J Exp Med (2020) 217:e20201012. 10.1084/jem.20201012 32926097 PMC7488867

[172] Ladikou EE Sivaloganathan H Milne KM Arter WE Ramasamy R Saad R Von Willebrand factor (vWF): marker of endothelial damage and thrombotic risk in COVID-19? Clin Med (2020) 20:e178–82. 10.7861/clinmed.2020-0346 PMC7539718 32694169

[173] Goshua G Pine AB Meizlish ML Chang CH Zhang H Bahel P Endotheliopathy in COVID-19-associated coagulopathy: evidence from a single-centre, cross-sectional study. Lancet Haematol (2020) 7:e575–82. 10.1016/S2352-3026(20)30216-7 PMC7326446 32619411

[174] Tiscia GL Favuzzi G De Laurenzo A Cappucci F Fischetti L di Mauro L Reduction of ADAMTS13 Levels Predicts Mortality in SARS-CoV-2 Patients. TH Open (2020) 04:e203–6. 10.1055/s-0040-1716379 PMC7456602 32879905

[175] Escher R Breakey N Lämmle B ADAMTS13 activity, von Willebrand factor, factor VIII and D-dimers in COVID-19 inpatients. Thromb Res (2020) 192:174–5. 10.1016/j.thromres.2020.05.032 PMC7245313 32505009

[176] Xie X Shi Q Wu P Zhang X Kambara H Su J Single-cell transcriptome profiling reveals neutrophil heterogeneity in homeostasis and infection. Nat Immunol (2020) 21:1119–33. 10.1038/s41590-020-0736-z PMC7442692 32719519

